# Anticipation of wheelchair and rollerblade actions in spinal cord injured people, rollerbladers, and physiotherapists

**DOI:** 10.1371/journal.pone.0213838

**Published:** 2019-03-15

**Authors:** Michele Scandola, Salvatore Maria Aglioti, Renato Avesani, Gianettore Bertagnoni, Anna Marangoni, Valentina Moro

**Affiliations:** 1 NPSY-Lab.VR, Department of Human Sciences. University of Verona, Verona, Italy; 2 Department of Psychology, University of Rome “Sapienza”, Rome, Italy; 3 Department of Rehabilitation, Sacro Cuore—Don Calabria Hospital, Negrar (VR), Italy; 4 Department of Functional Recovery and Rehabilitation, “San Bortolo” Hospital, Vicenza, Italy; Stevens Institute of Technology, UNITED STATES

## Abstract

Embodied Cognition Theories (ECT) postulate that higher-order cognition is heavily influenced by sensorimotor signals. We explored the active role of somatosensory afferents and motor efferents in modulating the perception of actions in people who have suffered a massive body-brain disconnection because of spinal cord injury (SCI), which leads to sensory-motor loss below the lesion. We assessed whether the habitual use of a wheelchair enhances the capacity to anticipate the endings of tool-related actions, with respect to actions that have become impossible. In a Temporal Occlusion task, three groups of participants (paraplegics, rollerbladers and physiotherapists) observed two sets of videos depicting an actor who attempted to climb onto a platform using a wheelchair or rollerblades. Three different outcomes were possible, namely: a) success (the actor went up the step); b) fail (the actor stopped before the step without going up) and c) fall (the actor fell without going up). Each video set comprised 5 different durations increasing in complexity: in the shortest (600ms) only preparatory body movements were shown and in the longest (3000ms) the complete action was shown. The participants were requested to anticipate the outcome (success, fail, fall). The main result showed that the SCI group performed better with the wheelchair videos and poorer with rollerblade videos than both groups, even if the physiotherapists group never used rollerblades. In line with the ECT, this suggests that the action anticipation skills are not only influenced by motor expertise, but also by motor connection.

## 1. Introduction

Anticipating the final phase of actions performed by others allows one to understand their intentions, synchronise one’s own behaviour and respond appropriately. Related to this process is the remarkable ability of the human brain to complete missing visual [1,2] and motor [3,4] information. Studies indicate that specific anticipatory “resonant” mechanisms enable an efficient prediction of the consequences of an observed action [5–10]. Both correlational [3] and causative studies in healthy [8,11,12] and brain damaged [13,14] people indicate that the discrimination and anticipatory coding of actions relies on a fronto-parietal ‘mirror’ network that appears to be particularly active with familiar as compared to unfamiliar actions [3,7,9,15–18]. This direct link between cognitive functions and the motor system provides substantial support to Embodied Cognition Theories [19,20] according to which cognitive processes are based on sensory, motor and emotional processes that in turn are grounded in body morphology and physiology [21]. In this vein, even higher-order cognition is heavily influenced by the experience of having a body with a specific shape or with specific sensory-motor capacities and of having specific interactions with objects and people in the environment [22]. It is widely held, for example, that children build fundamental concepts (e.g. representations of space and time) by means of perception and action. Actions involving spatial exploration or the exploration of objects also have a mediating role in cognitive development [23].

Studies of domain-specific motor expertise in adults show that athletes exhibit superior abilities in motor imagery [24] and in predicting the outcome of observed actions [7,25]. For example, expert basketball players can predict the outcome of free shots seen in a video earlier and more accurately than individuals with comparable visual experience but without motor expertise (i.e. coaches or sports journalists). This advantage is particularly evident in the first phases of actions, for example, when the ball has not yet been thrown and the player is bending his/her knees to prepare for the shot. Thus, prediction skills are not so much associated with the visual anticipation of the trajectory of the ball but rather with the players’ ability to ‘read’ the body kinematic by means of an internal motor simulation of the same action [7]. It is also significant that both long- and short- term physical training programs can increase people’s ability to predict the outcome of others’ actions [10].

The study of people suffering from a complete or incomplete massive body-brain disconnection, such as for example, spinal cord injured (SCI) patients, provides a potentially important contribution to understanding the link between higher-order cognition and somatosensory and motor processing. Tellingly, spinal cord lesions do not change the physical shape of the body, but dramatically alter the motor and sensory functions in the body parts below the lesion level. Thus, SCI participants may represent an ideal model for exploring the degree to which sensorimotor deficits alter cognitive functions. In fact, in this population, all the post-lesional changes in cognitive functions do not depend on brain damage or modifications in their body form but are exclusively related to the loss of sensory-motor afferences from and efferences to the disconnected body parts. Recent studies in SCI people conducted by our own and other research groups indicate post-lesional modifications in body representations [26–32] and motor imagery [33,34]. In addition, a reduction in the representation of peripersonal space around the feet in paraplegics has been documented [34]. Crucially, these deafferentation/deefferentation related changes are topographically specific, involving the paralyzed body parts and sparing the non-disconnected body parts [34,35].

A reduced capacity of paraplegics to discriminate biological motion (e.g. the direction in which a point-light walker moves) has also been reported [36]. Using a matching-to-sample task requiring the participants to discriminate body form and body actions, we found that paraplegics did not perform as well as the healthy controls specifically when paralyzed lower body parts were involved [37]. Significantly, it has also been shown that paraplegic people who practice sport were better at discriminating actions executed using upper body parts with respect to those who did not practice a sport, suggesting that specific new expertise may develop after a SCI [37]. In contrast, Bloch and colleagues [38] have reported a deficit in implicit learning of motor sequences in SCI people. In this experiment, two groups of paraplegics and healthy participants had to follow the instructions given by a computer that indicated which keyboard button in a series had to be pressed. The sequence of buttons to press was not random and was repeated for a certain number of times, in order to allow implicit motor sequence learning. Participants were asked to respond as fast as possible. Results showed that differently from controls, SCI did not increase their performance with the repetition of the sequence, hinting at a specific implicit procedural learning deficit. Thus, the debate on the possibility that motor deafferentation and deefferentation can impair the acquisition of novel motor expertise is still unsettled.

In the present study, we explored whether the expertise in using a wheelchair acquired by SCI people influences their abilities to anticipate actions. To address this issue, we devised a Temporal Occlusion paradigm involving paraplegic participants with long standing wheelchair experience, physiotherapists with visual but not motor expertise with wheelchairs, and healthy rollerblade experts. Participants were requested to observe two series of videos, one depicting an actor attempting to climb onto a platform using a wheelchair and one with an actor performing the same action but with rollerblades. Crucially, these stimuli allowed us to investigate in SCI the post-lesion learning of new skills.

The participants were asked to predict the final phase of the action in each video by selecting one out of three possible alternatives: i) the actor completes the action successfully; ii) the actor is not able to climb onto the platform or iii) the actor falls. This last condition, allowed us to explore also the potential role of defensive reactions in action anticipation.

Defensive processes towards dangerous/noxious stimuli have been demonstrated to have a role in modulating attention [39–41] and in the embodiment processes [42–44]. For example, the tendency to include the other person in one’s own representation is influenced by positive or negative interpersonal attitudes which may derive from both short-term individual interactions or consolidated socio-cultural stereotypes [42]. In addition, unsuccessful action attempts with concomitant error analysis can modulate the degree of action error awareness in patients suffering from anosognosia for hemiplegia [45]. In a similar vein, a potentially dangerous stimulus can modulate the expression of symptoms in neurological conditions, for example with a partial recovery of awareness in body representation disorders [44,46] or a reduction in involuntary movements in the anarchic hand syndrome [45]. Crucially, these modulations occur in implicit way, as potentially dangerous stimuli activate the ortho-sympathetic system [47] which impacts on motor and cognitive responses enhancing “fight and escape responses”.

Two control groups were involved in the study. The physiotherapists took part in the experiment in order for us to be able to ascertain whether pure visual expertise originating from them having worked with people in wheelchairs would play a similar role to the sensory-motor expertise that characterises SCI people. The rollerbladers (who had a great deal of experience with rollerblades but were inexperienced in the use of wheelchairs) took part in order for us to compare the role of expertise on action anticipation in healthy vs. deafferented/efferented people.

The following hypotheses were made: i) if somatosensory experience can modify action anticipation abilities even after lower limb deafferentation/deefferentation, then paraplegics should perform more accurately with the Wheelchair videos and rollerbladers should perform better with the Rollerblade videos; the performance of the physiotherapists should be average with both videos; ii) if neuroplasticity after SCI compromises the ability to build new action anticipation abilities, then paraplegics should not perform more accurately with the Wheelchair videos than the rollerbladers and physiotherapists, while rollerbladers should perform better with the Rollerblade videos; and iii) if a more general familiarity with a particular tool is sufficient for the task to be performed adequately then both paraplegics and physiotherapists should perform better than the rollerbladers with the Wheelchair videos. Finally, iv) due to the impact of defensive processes towards dangerous stimuli, we expected more efficient anticipatory responses to be triggered by the videos in which falls were displayed by respect to emotionally neutral stimuli.

## 2. Materials and methods

### 2.1 Participants

36 participants, divided into three groups of 12 individuals (all native Italian speakers) participated in the experiment. The spinal cord injury group (SCI: all males, age = 39.7±14.36, see Table 1 for clinical details) was composed of participants who had suffered from a traumatic spinal cord injury leading to paraplegia. This condition is characterised by the complete absence of sensations and voluntary motricity in the below-lesion lower limbs with normal sensory-motor functioning of upper limbs. Another group was composed of physiotherapists (PHY: 5 males, age = 36±7.53) who had been working in the field of SCI rehabilitation for at least one year but who were not able to use rollerblades. Finally, skaters with at least three years of experience using rollerblades (SKA: 8 males, age = 34.4±8.02) constituted the third group. The sample size for the three groups was determined taking into account the difficulty in finding these particular participants, and the typical sample sizes used in this typology of studies [25]. Posterior power analysis was computed by means of simulation, using a moderate effect size for standardized coefficients (effect size = 0.25) [48] in the most complex interaction. A power of 0.83 was reached with this sample size.

**Table 1:** Clinical and demographic details of the SCI participants.

	Age	NLI [49]	SCIM-3 [50]	Months from lesion onset
P1	29	T8	75	78
P2	47	T5	75	20
P3	27	T4	68	32
P4	43	T9	71	26
P5	24	T4	73	16
P6	25	T4	35	293
P7	60	T5	62	13
P8	43	T10	74	22
P9	71	T4	72	97
P10	34	T3	71	11
P11	39	T1	67	91
P12	34	T7	72	11

Paraplegic participants’ clinical details regarding the level of lesion (NLI = Neurological Level of Injury, from C1 to S4), the completeness of lesion (AIS = Asia Impairment Scale = A, complete lesions) and the degree of autonomy in activities of daily life (SCIM-3 = Spinal Cord Independence Index- version 3, from 0 –absence of autonomy- to 100 –complete autonomy). All participants were right-handed males.

It is worth noting that the SCI and the PHY participants had no experience with rollerblades while they had a daily experience with wheelchairs. Apart from the difference due to the injury, the two main differences between these two groups were the following: i) the SCI group had motor experience with wheelchair use while the PHY group had an experience of the tool prevalently visual; ii) even if they were both naïve to rollerblade use, the PHY group could potentially use rollerblades, while for the SCI group this was impossible, due to the paralysis. All participants signed the consent form. The study was approved by the Ethics committee of the Province of Verona (Prot. N. 40378) and was conducted in accordance with the ethical standards of the 2013 Declaration of Helsinki. SCI participants were recruited on a voluntary basis thanks to the cooperation with the Spinal Units of the Sacro Cuore Hospital (Negrar, Verona) and the San Bortolo Hospital (Vicenza), that are part of the International Group for Research into Spinal cord injury (http://profs.formazione.univr.it/npsy-labvr/spinal-cord-injury-research-center/).

### 2.2 Stimuli

Two series of videos were recorded (Canon EOS 600D Video Camera, 640x480, frame rate: 40 fps). These showed a paraplegic person in a wheelchair or a healthy rollerblader while they were in the act of climbing onto a wooden platform. The videos were recorded in the same room using the same equipment. The height of the platform simulated a standard pavement (30 cm). In this way, the action involving the wheelchair reproduced a very frequent situation in the everyday life of paraplegics comprising a motor activity that they are barely trained for in rehabilitation. For this reason, for the purposes of the experiment, we considered that the wheelchair action was very familiar for the SCI and PHY groups, with the difference being that the former had specific motor experience and therefore expertise, while the latter only had visual expertise and knowledge of kinematics. The video of the rollerblader served as a control since it involved an action which was very similar in terms of aims but very different in terms of kinematics and type of required motor expertise.

The series of videos showed a male actor (either in a wheelchair or wearing rollerblades) performing the same action. In each series, there were three different possible endings with the individual: i) Success: successfully climbing up onto the platform; ii) Safe fail: failing to complete the action but without any other consequences or iii) Fall: failing in the attempt to perform the action and falling to the ground.

**Fig 1:**
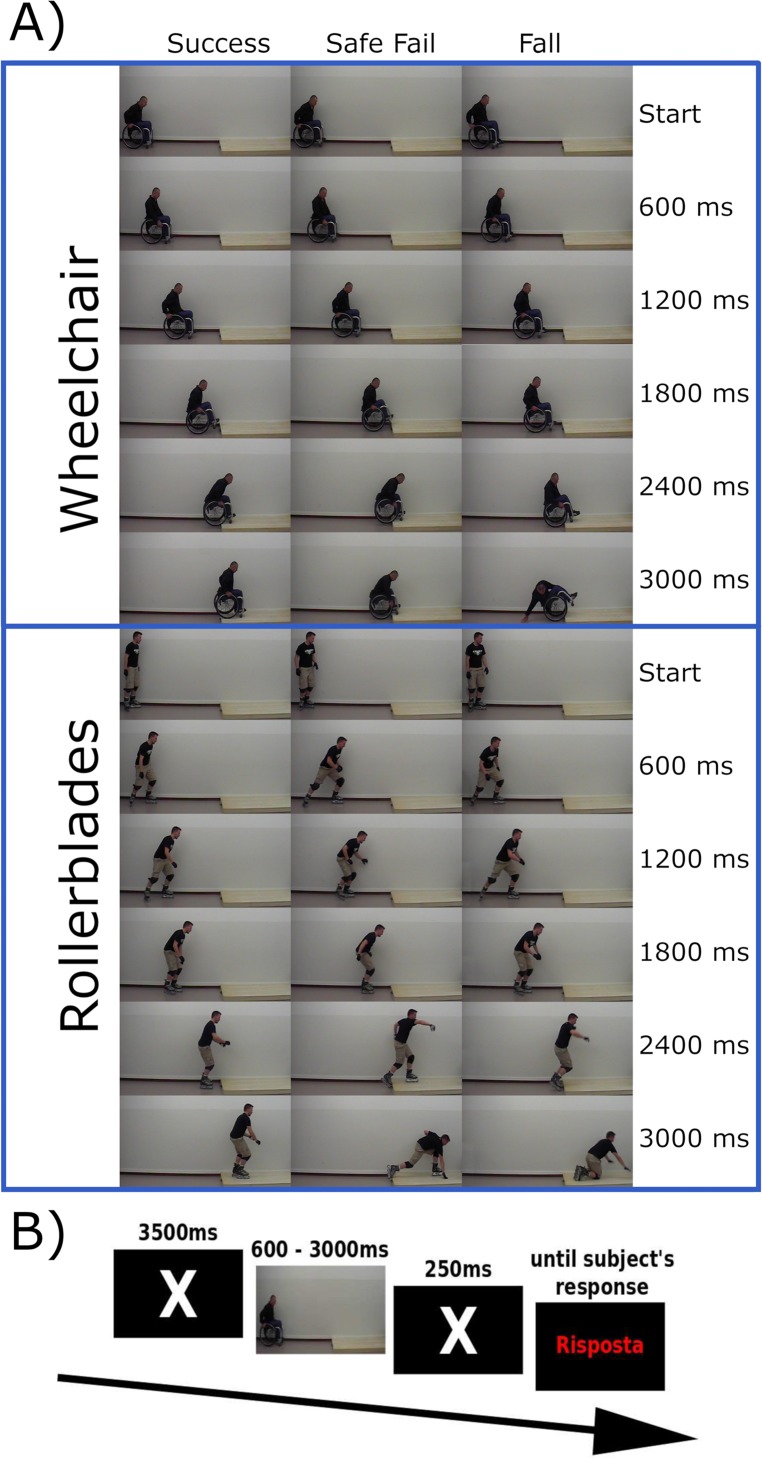
Stimuli and procedure. A) In the upper part the Wheelchair videos at different durations (Start, 600, 1200, 1800, 2400, 3000 ms) in the three endings, while in the lower part the Rollerblade videos. B) Trial timeline.

From among the 68 videos recorded, we selected the 6 most natural videos for each Tool (wheelchair or rollerblades) and each Ending, for a total of 36 videos. The duration of the videos was edited so that they all lasted 3 seconds and the colours were levelled out using GIMP software (ver. 2.8.18, www.gimp.org). Finally, the videos were divided into 5 clips, each of them starting from the beginning but with various different durations (600ms, 1200ms, 1800ms, 2400ms or 3000ms at 40 fps) for a total of 180 clips (see Fig 1A). After the execution of the experimental task, a group of 20 participants (11 SCI, 9 healthy controls) who were not recruited in the first action anticipation study were asked to rate along visual analogue scales the valence of the different stimuli. One SCI participant was excluded because he had been a professional rollerblader before the lesion onset. The ratings concerned the degree of anxiety, arousal, unpleasantness and unexpectedness induced by each video. These analyses showed that Fall and Safe fail endings elicited more negative emotions (anxiety and unpleasantness) than Success endings, while neutral emotions (arousal and unexpectedness) were more elicited by Success endings than Safe fail and Fall endings. In addition, Wheelchair videos were related to higher negative evaluations than Rollerblades videos. More detailed data are reported in Section A in S1 File (Table A.1, Code A.1, Table A.2).

In each video, the actors started from the same point and covered the same distance to reach the same platform; the duration of the videos was kept constant.

Kinematic analysis was conducted on the videos in the frames at 0, 600, 1200 and 1800 ms, taking into account the distances covered, the right elbow angle, and the left knee angle with respect to the floor (see Section B in S1 File for further information, Figure B.1, Table B.1, Code B.1, Table B.2, Code B.2, Table B.3, Table B.4).

The kinematics of the 6 videos for each tool and ending was not different (see Table B.3 in S1 File). Nevertheless, in both rollerblades and wheelchair videos the velocity was faster for Success than other endings. As expected, the angle of the right elbow was different for rollerblade and wheelchair videos and for different endings (see Table B.4 in S1 File).

### 2.3 Procedure

The participants were seated in a comfortable position at a distance of about 60 cm from the screen and were asked to remain as still as possible while they were performing the Progressive Temporal Occlusion task [5].

Each screening began with a white fixation cross on the screen (3500ms) and then the video was shown for a variable duration (from 600ms to 3000ms). After this, a new fixation cross was shown for 250ms, followed by the word “RISPOSTA” (the Italian word for “answer”) in red text. At this point, the participants were requested to verbally predict how they thought the video would end. The text on the screen remained until the participant provided a verbal response (Fig 1B), choosing from among 3 possibilities: i) “Riesce” (“Success”); ii) “Salvo” (to indicate “Safe fail”) for actions which failed but were without consequences and iii) “Cade” (“Fall”). These three words were selected because they are similar in length in Italian.

The experiment was divided into four blocks with pauses in between each block (three pauses in total). The participants observed one of the series of videos (i.e. involving either the Wheelchair or the Rollerblades) in the first two blocks while in the second two blocks the Tool changed. The order of the pairs of blocks was counterbalanced across participants. In addition, within each individual block the order of the videos was randomized and each video was shown twice. There was the same number of videos (36) for each duration (5) and for each ending (3 for wheelchair and 3 for rollerblades). Each video was shown twice, for a total of 90 videos per block and 360 videos in the entire experiment.

Before the task, two example videos were shown in order to illustrate the task and the three possible endings. These featured a girl on rollerblades and a boy in a wheelchair while they were making three attempts (one for each ending) to climb up onto a platform. The actions were analogous to the actions used during the experiment, but the environment and the people involved were different. Furthermore, these clips were not divided into different durations but shown in their entirety.

The duration of the whole experiment, introduction and pauses included, was about 80 minutes.

### 2.4 Data handling and statistical analysis

The scores were aggregated for Participant, Group, Length (600ms, 1200ms, 1800ms, 2400ms, 3000ms), Tool (Wheelchair, Rollerblades) and Ending (Success, Safe fail, Fall). As motor expertise had a stronger impact on action anticipation when people observed the earlier as compared to the later phases of the videos (early occlusion conditions), data concerning the first three durations of the clips (600ms, 1200ms and 1800ms) were analysed separately from those pertaining to the longer clips (2400ms and 3000ms). In effect, in the longer clips the actions were shown almost in their entirety meaning that participants executed a task of Action Recognition rather than Action Anticipation in these cases.

We used a Bayesian approach that allowed us not to incur in p-values related controversies about the replicability of results [51–54].

The results of Bayesian Analyses produce posterior distributions, computed from a prior distribution (representing the hypothesis to be evaluated) and the likelihood distribution of data [55,56]. The use of a non-informative prior (i.e. with large variances and means distributed around zero) prevents results being biased towards alternative hypotheses [57] and respects the Laplacian principle of indifference [58].

In order to perform the Bayesian analysis with R [59], the jagsUI ver. 1.4.9 [60] package was used to connect it to JAGS ver. 4.0 [61]. This is a GNU software used to perform Markov Chain Monte Carlo (MCMC) simulations via the Gibbs sampling algorithm [62].

All the analyses considered these independent categorical variables: Group (SCI, SKA, PHY), Tool (Wheelchair, Rollerblades) and Ending (Safe fail, Succeed, Fall) as fixed factors; Length of time was a continuous independent variable, centred and scaled in the [-1; 1] range, and Participants was a categorical random intercept. Within the model all the interactions between independent variables were considered. We estimated the posterior distributions of all the levels of the independent variables (Group, Tool, Length, Ending) and their interactions. These posterior distributions were used to compute the contrasts among the levels of the independent variables of interest (i.e. joint posterior distributions of differences of the coefficients of regression– β –of the linear part of the analysis), by means of Hierarchical Bayesian Logit models [56,63,64] with a non-informative prior (see Section C, Table C.1 and Code C.1 in S1 File for details).

The analyses were focused on the contrasts (i.e., joint posterior distribution of the differences) among the μ values of the coefficients of regression βs, with each of them representing a different level of the independent variables or combinations of levels of interaction.

The contrasts are reported as the Mode (Mo), which is a measure of central tendency, and the 89% Highest Posterior Density Interval (HPDI), that is the Bayesian analogue of the frequentist Confidence Interval; more precisely, this is the narrowest interval whose underling curve contains the 89% of the area [65]. For all the contrasts we also computed the Effective Sample Size (ESS) and the R^ values. The ESS is the division between the actual sample size and the amount of autocorrelation (i.e., the correlation of series of data with themselves) within the posterior distributions, and it is a measure of how many independent samples we have obtained [56]. It represents an index of accuracy of the MCMC with higher values indicating better accuracy (ESS > 10000) [56]. The R^ values (also known as the Gelman’s Diagonal) [66,67] indicate convergence of the MCMCs when they are within [1 ÷ 1.1). In order to reach convergence and accuracy, a total of 26000 samples for the 600-1800ms data, and 76000 for the 2400–3000 data were drawn after 1000 adapt and 1000 burn-in samples for each of 5 chains, for a total of 125000 samples for the 600-1800ms data and 355000 samples for the 2400-3000ms data. In all the cases the ESS of the contrasts are at least 10000, and the R^ are within [1 ÷ 1.1).

Here the contrasts are considered as indicating a “credible” difference between two variables when the HPDI is completely greater or completely lower than the Region of Practical Equivalence (ROPE) [56]. In fact, ROPE indicates a band of values that are equivalent to the null value for practical purposes [56]. Our ROPE range is between [-0.02; 0.02], as previously used in similar statistical analyses [68]. In order to accept that two distributions are credibly different, the HPDI on their difference must be entirely lower than −0.02 or greater than 0.02 and thus out of the ROPE. All the HPDIs that are not within these criteria are non-credible differences (i.e. indicating neither a presence nor an absence of difference). In reporting the results we will use the terms “credible” and “non-credible” as in use in Bayesian statistics.

The whole set of results is shown in Section D, Table D.1, Table D.2, Table D.3, Table D.4, Table D.5, and Table D.6 in S1 File).

Goodness of fit of models was tested by means of visual inspection of the cumulated posterior distributions of fixed effects, without the contribution of covariates or random effects, with respect to the actual data representation. Graphical representation is reported in Fig. C.1 in S1 File.

## 3. Results

### 3.1 Video clips from 600 to 1800 ms: Action anticipation

HPDI comparisons show the presence of differences outside the ROPE for the main effects of Ending, for the Group:Tool, Group:Ending and Tool:Ending interactions, while no differences among covariates are outside the ROPE. The complete results for the covariates are reported in Table D.2 in S1 File.

#### 3.1.1 Main effects

The only credible main effect resulting from the analysis regards the Ending. In fact, the performances for Success endings are better than for Fall (Mo = -1.67, HPDI = -1.77, -1.57) and Safe fail endings (Mo = -0.52, HPDI = -0.61, -0.43), while the performance for Safe fail endings are better than for Fall endings (Mo = 1.16, HPDI = 1.05,1.26).

#### 3.1.2 Group: Tool interaction

The SCI group shows worse performance in Rollerblade videos than the SKA (Mo = -0.16; HPDI = -0.26, -0.16) and PHY groups (Mo = -0.096; HPDI = -0.19, -0.03). Conversely, the SCI group shows better performance than the other two groups in Wheelchair videos (against SKA: Mo = 0.16; HPDI = 0.05, 0.26; against PHY: Mo = 0.10; HPDI = 0.03, 0.19). The comparisons of the performance in the SKA and PHY groups show marginal but not credible differences (i.e., the HPDIs contain the zero: Rollerblade: Mo = 0.06; HPDI = -0.03, 0.15; Wheelchair: Mo = -0.06; HPDI = 0.03, -0.15).

Finally, the SCI group shows better performance for Wheelchair than Rollerblade videos (Mo = -0.16, HPDI = -0.28, -0.06)-. Conversely the SKA group have a better performance for the Rollerblade videos (Mo = 0.14, HPDI = 0.03, 0.26), while the PHY group does not show differences (Mo = 0.02, HPDI = -0.08, 0.13).

#### 3.1.3 Group: Ending and tool: Ending interactions

The PHY group has better performance than the SCI and the SKA groups in Safe fail and Fall endings (SCI vs. PHY in Safe fail endings: Mo = -0.29, HPID = -0.42, -0.16; PHY vs. SKA in Safe fail endings: Mo = 0.36, HPDI = 0.23, 0.50; SCI vs. PHY in Fall endings: Mo = -0.18, HPDI = -0.33, -0.03; PHY vs. SKA in Fall endings: Mo = 0.22, HPDI = 0.07, 0.37), while in Success endings both these groups have a better performance than the PHY group (SCI vs. PHY in Success endings: Mo = 0.47, HPDI = 0.33, 0.60; PHY vs. SKA in Success endings: Mo = -0.59, HPDI = -0.72, -0.45). The Fall ending is easier in Rollerblade than Wheelchair videos (Mo = 0.17, HPDI = 0.05, 0.30) and Success endings in Rollerblade videos (Mo = 0.15, HPDI = 0.04, 0.25). In Wheelchair videos, Success endings are easier than Fall endings (Mo = -0.15, HPDI = -0.25, -0.04).

All the other comparisons do not exclude the ROPE.

**Fig 2:**
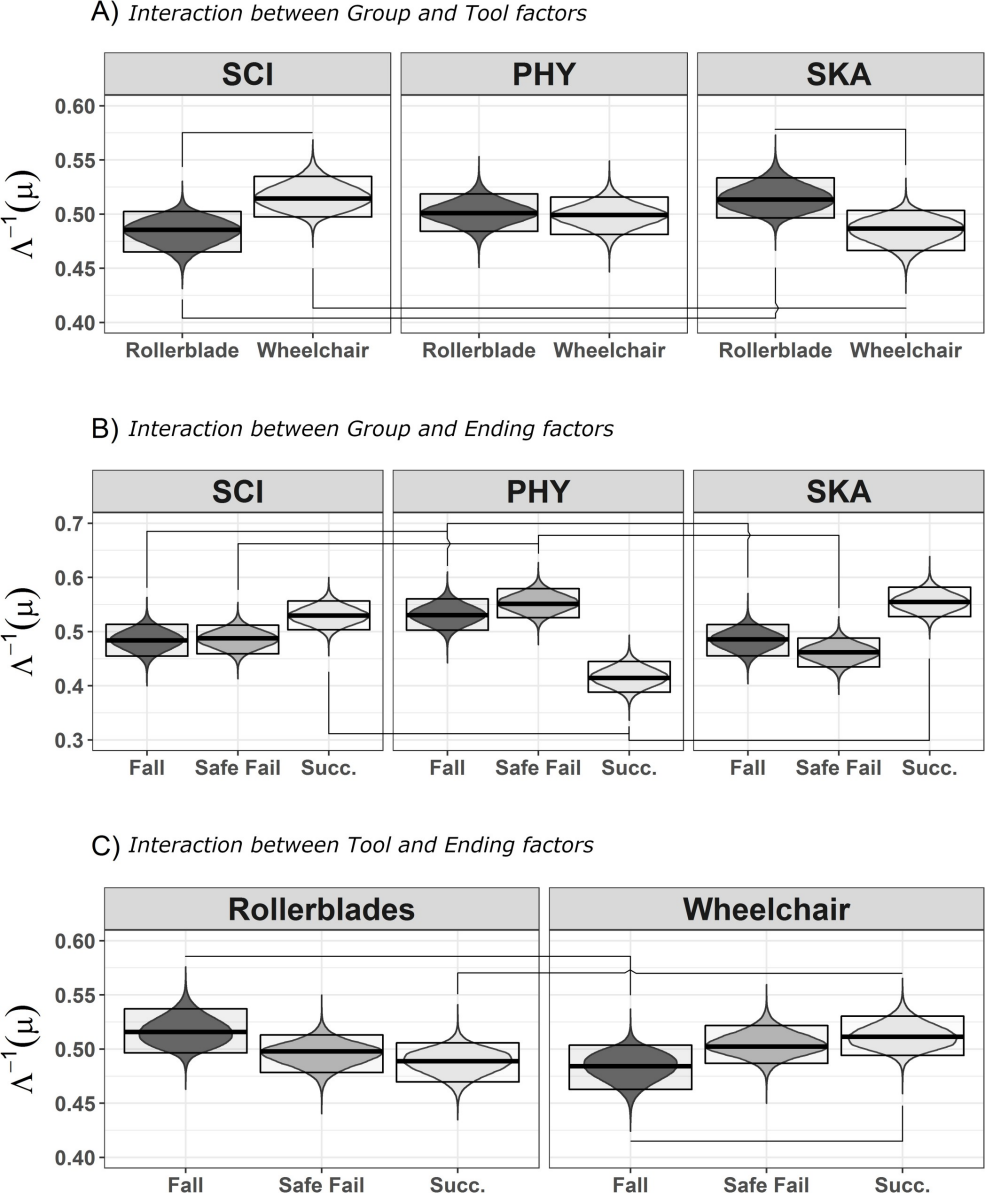
Graphical representation of the joint posterior Bayesian distributions regarding responses to the videos between 600 ms and 1800 ms. Only the interactions that show at least one statistically credible difference are represented. A) interaction between Group and Tool factors. B) the interaction between Group and Ending factors. C) interaction between Tool and Ending. The posterior distribution values were transformed by means of the inverse logit function (Λ^-1^). All posterior distributions whose HPDI (highest posterior density interval) of the difference is completely outside the ROPE (region of practical equivalence) are connected by a line. Violin plots represent the distribution of the posterior distribution, while the box limits are the HPDI and the central darker horizontal line is the mode of the posterior distribution. SCI = spinal cord injured people; PHY = physiotherapists; SKA = skaters; Succ. = Success.

See Fig 2 for a graphical representation of these results.

### 3.2 Video clips from 2400 to 3000 ms: Action recognition

HPDI comparisons show credible differences (i.e., outside the ROPE) for the main effects and the covariate Length alone, and for the Group:Tool, Group:Ending, Tool:Ending and Group:Tool:Ending interactions.

#### 3.2.1 Main effects and Length covariate

Performance for Rollerblade videos are better than for Wheelchair videos (Mo = 1.83, HPDI = 1.52, 2.22) and in general the SKA and the PHY groups have better performance than the SCI group (Mo = -0.81, HPDI = -1.47, -0.20; Mo = -0.73, HPDI = -1.34, -0.12, respectively). In addition, in these longest frames the effect of the Length is clear (Mo = 0.46, HPDI = 0.04, 0.89), suggesting that the last, longest frame is easier than the previous one.

#### 3.2.2 Group: Tool interaction

As in the shortest durations, the SCI group shows better performance in Wheelchair than Rollerblade videos (Mo = -0.53, HPDI = -0.93, -0.16). Moreover, the SCI group shows better performance in Wheelchair videos than the PHY and SKA groups (Mo = 0.38, HPDI = 0.09, 0.74; Mo = 0.39, HPDI = 0.05, 0.77, respectively). *Viceversa*, the SCI group have worse performance in Rollerblade videos than the PHY (Mo = -0.38, HPDI = -0.74, -0.09) and SKA groups (Mo = -0.39, HPDI = -0.77, -0.05).

The comparisons between the SKA and the PHY group do not exclude the ROPE.

#### 3.2.3 Group:Ending, Ending:Tool and Group:Tool:Ending interactions

The SCI group has better performance with Success than Safe fail endings (Mo = -0.42, HPDI = -0.87, -0.05) while the PHY group has better performance with Safe fail than Success endings (Mo = 0.43, HPDI = 0.02, 0.89). In Success endings the SCI group also has better performance than the PHY group (Mo = 0.53, HPDI = 0.16, 0.97).

In particular, the SCI group shows better performance for Fall and Success than Safe fail endings for Rollerblade videos (Mo = -0.43, HPDI = -0.85, -0.05; Mo = -0.73, HPDI = -1.20, -0.30, respectively), while for Wheelchair videos the Safe fail ending has the best performance (against Success: Mo = 0.73, HPDI = 0.30, 1.20; against Fall: Mo = 0.43, HPDI = 0.05, 0.85). For Safe fail endings this group has better performance for Wheelchair than Rollerblade videos (Mo = -0.77, HPDI = -1.31, -0.29). Conversely, the SCI have better performances with Rollerblade videos than Wheelchair videos (Mo = 0.66, HPDI = 0.20, 1.17) with Success endings.

Accuracy for Fall and Safe fail endings is higher for Rollerblade videos than Wheelchair videos (Mo = 1.03, HPDI = 0.59, 1.53; Mo = 1.53, HPDI = 1.10, 2.00, respectively); however, Success endings have better accuracies in Wheelchair than Rolleblade videos (Mo = -2.61, HPDI = -3.03, -2.20).

Performance for Success endings in Wheelchair videos are better than for Fall (Mo = -2.08, HPDI = -2.45, -1.72) and Safe (Mo = -1.80)

The better SCI’s performance in Safe fail endings of Wheelchair videos is observable also when these are compared to the PHY and SKA groups (Mo = 0.64, HPDI = 0.20, 1.08; Mo = 0.52, HPDI = 0.11, 0.99, respectively). However, for Rollerblade videos the SCI group shows better accuracy for Success endings than the PHY and SKA group (Mo = 0.38, HPDI = 0.03, 0.78; Mo = 0.58, HPDI = 0.16, 1.06, respectively).

The only result from other groups come from the SKA group who has better performance in Success endings of Wheelchair than Rollerblade videos (Mo = -0.54, HPDI = -1.04, -0.03).

In conclusion these analyses show that a better ability in recognizing Success endings in Wheelchair videos, and risky (Fall and Safe) endings in Rollerblade videos. The SCI group exhibited better accuracy than the PHY group in Success endings, and worse accuracy in Safe fail endings.

Furthermore, the SCI group shows a more prudential approach with Wheelchair videos than with Rollerblade videos, with better performances with “Safe fail” endings than the other endings; while the SKA group recognizes “Success” endings with a better performance in Wheelchair than in Rollerblade videos.

**Fig 3:**
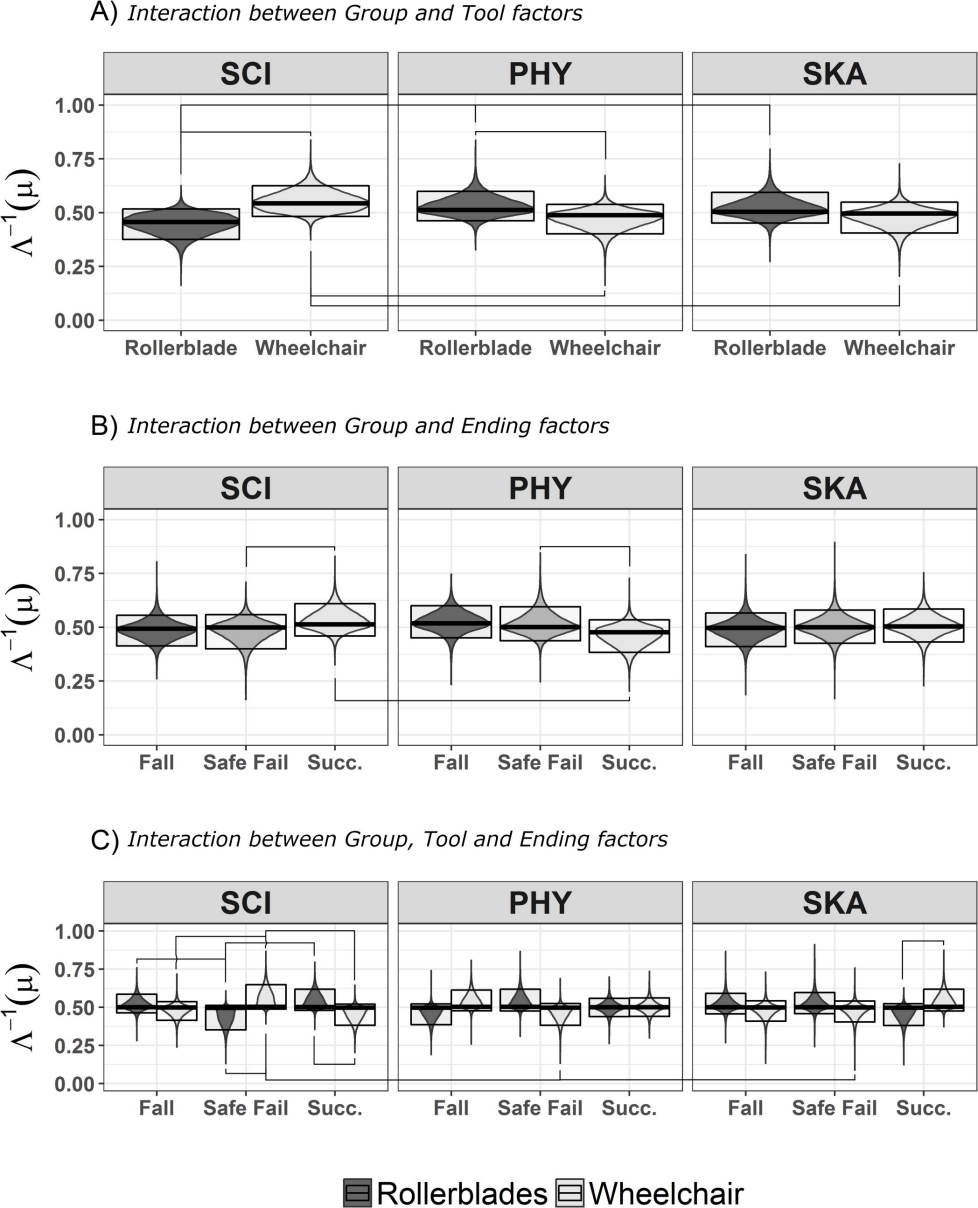
Graphical representation of the joint posterior Bayesian distributions regarding responses to the videos between 2400 ms and 3000 ms. The posterior distributions represented are the interactions that show at least one statistically credible difference. A) the interaction between Group and Tool factors. B) Interaction between Group and Ending factors C) Interaction between Group, Tool and Ending factors is represented. Description as in Fig 2. SCI = spinal cord injured people; PHY = physiotherapists; SKA = skaters; Succ. = Success.

Results are graphically reported in Fig 3.

## Discussion

In this study, we used a Temporal Occlusion paradigm [69] to investigate in SCI participants the role of new, post-lesional motor expertise and defensive components in the ability to anticipate the endings of actions. Based on the notion that expertise improves action anticipation skills [7], we explored the degree to which SCI participants with massive body brain deafferentation/deefferentation are able to predict the outcome of actions involving wheelchairs (which they have become familiar with from both a perceptual and motor point of view after their lesion) vs actions involving rollerblades (which are currently impossible and not performed before the lesion onset). This allowed us to investigate the effects of lesion-correlated new motor learnings in action discrimination. A group of physiotherapists who are expert in the rehabilitation of SCI patients was used to dissociate the effects of mere perceptual expertise with wheelchairs, and a group of expert rollerbladers permitted to control for the effects of different domain-specific expertise.

We expected to replicate the results on effects of expertise in action anticipation [7], and therefore to find that the SKA and SCI groups perform better than the other groups in the Rollerblade and Wheelchair videos, respectively (Hypothesis i, as described in Introduction). However, if the impact of SCI completely impairs the action anticipation ability, we should observe that the SCI group have worse performances than the other groups in both Wheelchair and Rollerblade videos. Finally, if the deficit in action anticipation is strictly related to sensory-motor deficits in the lower part of the body and to the impossibility to perform lower limb actions, we should observe that SCI performance in Rollerblade video (but not in Wheelchair videos) is worse than PHY ones (Hypothesis ii). In effect although PHY are naïve to skating, their preserved sensori-motor functions could guarantee a correct anticipation of action in this condition (Hypothesis ii). However, if the familiarity with tools were sufficient, the performance of both PHY and SCI groups should be comparable in Wheelchair videos, and better than the SKA group (Hypothesis iii). Finally, the effect of endings which elicit a defensive reaction (i.e. Fall and Safe fail) is expected to impact the performance. Indeed, if endings eliciting defensive responses recall more attentional resources or activate some forms of alert reactions, we expect that in these endings the responses are more accurate (Hypothesis iv), in particular in the groups where the seen actions are more familiar and thus likely more embodied.

SCI participants performed better with the Wheelchair videos and worse than both PHY and SKA participants with the Rollerblade videos. Furthermore, SKA participants performed better with the Rollerblade videos and worse than SCI participants with wheelchair videos. This double dissociation was particularly evident for the videos of shorter lengths of time (600ms, 1200ms, 1800ms). Interestingly, however, also in the longest videos the SCI group performed better with the Wheelchair videos, and worse with the Rollerblades videos, than the other two groups (2400ms– 3000ms). Overall, the results suggest that in order to carry out the task, SCI participants accessed to their specific motor expertise not only in action discrimination (as shown by their performance with Wheelchair videos) but also in the action anticipation system (as shown by their performance in the shortest videos). In addition, the differences between the three groups indicate that the effects of motor expertise are very specific and do not generalize to actions which are not present in the individual’s motor knowledge. This result is in contrast with Bloch and colleagues [38] who find a deficit in implicit learning of motor sequences in SCI people. In fact, our results indicate that SCI people are able to learn new motor procedures after the lesion onset (i.e. driving a wheelchair), that are very specific for their conditions and that are not present in the motor knowledge of healthy people. Moreover, our data show that these new abilities extend from sensory-motor to cognitive processes of action, inducing a task-specific ability to anticipate the final of the acquired actions.

There were no differences in the physiotherapists’ performance with regard to the tool used, indicating that visual knowledge about what is like using a wheelchair acquired as part of one’s work is not sufficient for developing specific abilities in action anticipation tasks. Nevertheless, the PHY group was better at identifying the “Fall” and “Safe fail” endings with respect to the other groups. This might be interpreted as an effect of their specific professional training, since they are required to pay close attention to patients due to the risk associated with their motor activity, a process that may lead PHY to anticipate patients’ falls and accidents. Conversely, both the SCI and SKA groups were better at anticipating “Success” endings.

### Embodied cognition and action anticipation

The ability to predict and anticipate the actions of other people is a largely implicit process that allows individuals to adapt their behaviour to various different situations and thus implement consistent responses. The effect of motor expertise in action anticipation has been demonstrated previously, in particular in the case of elite athletes [25,70–72] and musicians [8,9,73]. Such effects have been interpreted as due to “motor resonance” process that automatically activate the onlookers’ motor system during observation of highly learned and extensively practiced actions. Even when the effect of visual familiarity as result of watching movies is taken into account, neural activity is still higher in individuals who have had direct motor experience of the action they are observing [8,17].

The neural correlates of motor resonance and action anticipation have been identified as being located in the fronto-parietal action observation network (AON) [71,72]. Importantly, however, a fMRI study carried out by Abreu and colleagues [25] showed that during Action Anticipation tasks, expert athletes also exhibited greater neural activity than naïve individuals in body discrimination related regions (i.e. the extrastriate body area), probably due to their expert reading of the body kinematics involved in an action. Moreover, there was higher activation in the bilateral inferior frontal gyrus and in the right anterior insular cortex of these athletes when they made errors, hinting at a clear role of these structures in mapping action errors. Correct action prediction induced higher posterior insular cortex activity in experts and higher orbito-frontal activity in novices, suggesting that body awareness is important for performance monitoring in experts, whereas novices rely more on higher-order decision-making strategies [25].

There are several possible reasons why one would expect a reduction in action anticipation abilities after SCI. First of all, deafferentation and deefferentation of below-lesion body parts induce relative processes of neuroplastic remapping, involving the premotor and parietal cortices [74–79]. In addition, behavioural studies have shown relevant changes, not only in action discrimination [27,36] but also in, body representation [29,37] and awareness, with phenomena involving corporeal misperceptions and illusions [31,80,81] in motor imagery [34] and in the representation of space [35]. Nevertheless, Pernigo and colleagues [37] find that in front of a reduction in action discrimination of the lower limbs form and action, paraplegics who regularly practice sports increase their abilities to discriminate the upper body parts which are not affected by the lesion. It is thus plausible that along with the reduction of some capacities the new conditions in which the person acts in the world lead to the enhancement of highly practiced functions. Taken together, these studies give support to the Embodied Cognition Theories (ECT) according to which bodily information, and action in particular, is central in defining and modulating cognition [82,83]. The most radical position in ECT postulates that cognition emerges from “the recurrent sensorimotor patterns that enable action to be perceptually guided” [84]. Our study does not assess symbolic cognitive processes and thus cannot give support to this radical view. Nevertheless, our data confirm that at least some cognitive functions, such as the capacity to anticipate the result of an action, are directly related to sensory-motor capacities. In fact, although the results regarding the skaters are not totally new and only confirm previous data about the effects of motor expertise in athletes [7], the performance of the other two groups highlights three aspects that can be discussed within the theoretical framework of ECT. The first is that the impossibility to do actions with some body parts (i.e. legs) compromises also the cognitive functions related to the same body parts (the performance of the SCI group in Rollerblade videos, compared to the PHY group). This expands our previous results showing a similar, topographically organized process for other cognitive functions (i.e., peripersonal space representation and motor imagery) [34,37]. The second is that motor abilities acquired in adulthood and after the spinal cord lesion also impact cognitive functions (the performance of the SCI group in Wheelchair videos). Finally, and crucially for the ECT is that mere theoretical knowledge about an action (the performance of the PHY group in the Wheelchair videos) is not sufficient to anticipate the end of the action, a skill that requires motor experience instead.

Our results demonstrate that motor expertise acquired after a neurological lesion improves performance in action anticipation relating to the newly learned action. A similar improvement was also found in a study in which SCI paraplegic participants and healthy controls executed a matching-to-sample visual discrimination task of body form and actions involving lower (affected by lesion) but not upper (spared by lesion) limbs [37]. Our result was also in keeping with a study reporting that paraplegics had difficulties in visually perceiving point-light displays of human locomotion [36]. Tellingly, participants with SCI who regularly practice sports (and thus became experts at using their upper body parts) significantly improved in discrimination tasks involving upper body parts suggesting that the new expertise related to motor activity does not increase only body representation but expands to action representation and anticipation [37].

The performance of PHY deserves discussion. It is worth noting that we specifically only recruited PHY participants who had been working in a Spinal Unit for at least one year. This means that, in principle, they were considerably knowledgeable from a perceptual point of view regarding the kinematics relating to wheelchair use. Moreover, people working in this area are usually given training on how to move in a wheelchair and how to go up and down steps as they are required to teach these abilities to patients during rehabilitation programs. Despite all of this, the PHY group performance in the wheelchair videos was not as good as that of the SCI group. Thus, expertise gained from the observation of others using a wheelchair without a certain amount of first-hand practice is not enough to ensure enhanced action anticipation skills. This indicates that real motor practice is needed to achieve expertise in domain-specific action anticipation tasks. Taken together, these results support the Embodied Cognition theory and previous evidence showing that massive deprivation induces changes in higher-order cognitive functions. Our study also confirms that sensory-motor abilities may be acquired after deafferentation/deefferentation [34,35,37] and that this new motor expertise induces cognitive adaptations. Thus, in keeping with ECT our results confirm the link between body features (such as form, size and sensory-motor functions) and cognition. In particular, our data indicate that sensory-motor information is critical for remodelling cognitive functions not only during infantile development, but also across lifespan and even after neurological lesions.

### A defensive reaction to potentially dangerous actions?

The responses of the SKA and SCI groups in the shorter videos seem to indicate a sort of bias towards a positive ending. In fact, in the shorter videos, SKA and SCI groups performed better with “Success” endings than with “Safe fail” and “Fall” endings. In the longer videos this effect was only present with the Wheelchair videos. This result does not depend on a different difficulty of the stimuli since the PHY group seemed to perform better with the “Fall” videos than with the other endings. We interpret this result as linked to the fact that PHY are influenced by their specific training. In fact, rehabilitation is often associated with the risk of patients falls and thus physiotherapists are trained to devote particular attention to potentially dangerous situations so that these can be anticipated and prevented. In the longer videos, this effect was only present with the Wheelchair videos.

The bias concerning the videos with “Success” endings is not in agreement with our initial hypothesis according to which the SKA and SCI groups would perform better in the Safe fail and Fall endings because of the dangerous nature of these stimuli. This hypothesis was based on data in brain damaged patients who seemed to be (unconsciously) primed by noxious and potentially dangerous stimuli. For example, dangerous stimuli evoke a prototypical skin conductance response in patients affected by anosognosia for hemiplegia who deny their acquired contralesional paralysis [44]. In a similar vein, exposure to dangerous actions improves awareness [46]. Furthermore, exposure to dangerous stimuli reduced the number of anarchic movements in a patient affected by anarchic hand syndrome, a rare neurological condition characterised by seemingly purposeful, goal-directed hand movements which the person afflicted by the syndrome is not in control of [43]. This dangerous stimuli related modulation of motor behaviour indicates that the SCI group would be more sensitive to the videos involving a “Fall” ending with respect to the other endings. In contrast, a sort of bias towards positive endings (i.e. the videos with “Success” endings) was found.

Several non-mutually exclusive explanations may explain this result. The first is in keeping with the “perceptual defense” theory [85] according to which perceptual and cognitive processing are inhibited by negative stimuli. In other words, the presence of ambiguous stimuli (in terms of emotional valence) would make people more prone to choosing the most positive interpretation, just as the SCI group in our study. Although this mechanism may have influenced the responses of the SCI group in some way, our original hypothesis posited that all three groups would exhibit a similar trend towards a positive bias. A second explanation, in keeping with the “relevance hypothesis”, is that people’s attention is preferentially attracted by stimuli that are relevant to guiding action [86]. In this vein, it is possible that rollerbladers and spinal cord injured participants would pay more attention to cues implying the correct and successful execution of an action while physiotherapists would pay more attention to cues that are more relevant to what they need to know when dealing with SCI patients. Although plausible, this explanation does not account for the results relating to the longer videos where the “Fall” endings were easier than the Success and Safe fail endings. A third explanation takes into account the fact that the sight of a person in a wheelchair may evoke (mainly at the start of the action, i.e. in the case of the shorter videos) some “protective” strategies, at least in the SCI and SKA groups, that lead to an implicit avoidance of potentially negative endings. The potential impact of a different degree of familiarity with the three action endings deserves some discussion. In effect, it might be possible that “Success” actions are more familiar than “Fall” or “Safe fail” actions. However, SKA participants are used to see others falling down and fall down themselves in their sport activities. Furthermore, were familiarity strongly at play in determining the different performance of the different groups, SCI and PHY should respond similarly to Wheelchair videos as both of them are used to look at people driving wheelchairs. Instead, performance was better for SCI participants than PHY participants.

### The effect of the duration of the videos

Our results confirm that the ability to predict the outcome of an action occur in the early phases of the execution of the action, at least in action anticipation tasks based on temporal occlusion paradigms. Indeed, we found an effect of time (with longest videos leading to better action anticipation performance) on rollerblades and on “Safe fail” videos within 600 ms and 1800 ms, and on all videos between 2400 ms and 3000ms. The advantage in action anticipation tasks due to motor expertise is in fact evident in the very first phases of the observed actions. This is in keeping with studies showing that the human motor system is preferentially activated by the initial phases of an incomplete action, suggesting its role in the anticipatory simulation of future action phases [18].

The Action Observation Network (AON) includes not only visual, occipital and -temporal areas but also motor, fronto-parietal areas [87]. Our data show that a body-brain disconnection can indirectly impact on the AON, inducing an impairment in action anticipation for actions that are impossible and increased performance for those actions that have become usual for individuals.

## Conclusions

In conclusion, by means of a Temporal Occlusion Paradigm, the study demonstrates for the first time, that body deafferentation and deefferentation lead to changes not only in execution but also in representation (i.e. anticipation of endings) of actions. In fact, in judging the possible ending of rollerblade videos, the SCI group had worse results than skaters but also than physiotherapists, who were naïve for rollerblade actions but able to move their legs and walk. Our results also confirm the effects of motor expertise, with the SCI group performing better than the other groups in Wheelchair videos and the SKA group in Rollerblade videos. This indicates that changes in sensory-motor functions and constraints of body impact on high order cognitive functions, and supports the theoretical position of Embodied Cognition Theories. Finally, we show that action anticipation ability in SCI is impaired for lower-limbs related actions, but is increased for the actions learned after the lesion and that constitute a new, domain specific expertise. Therefore, in addition to post-lesional negative neuroplastic changes in body and space representations [35–37], we report evidence of potentially positive changes linked to new expertise. These changes may be particularly useful in the devising of new strategies for rehabilitation and in terms of optimising programs dedicated to the recovery of autonomy in everyday life after SCI.

## Supporting information

S1 File Supplementary information of: Anticipation of wheelchairs and rollerblades actions in spinal cord injured people, rollerbladers and physiotherapists (PDF)
