# Supplementary material for: Anticipation of wheelchair and rollerblade actions in spinal cord injured people, rollerbladers, and physiotherapists
Source: PLoS One. 2019 Mar 15;14(3):e0213838. doi: 10.1371/journal.pone.0213838 (PMC6420014; doi:10.1371/journal.pone.0213838)
Supplement: S1 File — (PDF) [file pone.0213838.s001.pdf]

Supplementary Information of: *Anticipation of wheelchair and rollerblade actions in spinal cord injured people, rollerbladers and physiotherapists*

Scandola M, Aglioti SM, Avesani R,Bertagnoni G, Maragoni A, Moro V.

A. The Emotional Valence of the Stimuli

Materials and Methods

Participants

10 participants suffering from spinal cord injury (3 females, NLI range: C5-T12, AIS: 4 A, 4 B and 2 C) were recruited at the Spinal Unit of the Sacro Cuore Hospital (Negrar, Verona). In order to avoid any effects of familiarity with the videos, none of patients was previously tested in the Action Anticipation paradigm.

Stimuli

The stimuli were the 36 videos used in the Action Anticipation paradigm (see the main text).

Procedure

The participants were seated at a distance of about 60 cm from the screen. They observed in a random order all the 36 videos for their entire duration (3000 ms each one). After each video participants had to evaluate along four 0 (minimal)-100 (maximal) VAS scales the degree of Anxiety, Unpleasantness (Negative emotions), Unexpectedness and Arousal (Neutral videos). The task lasted about 20 minutes.

Data handling and Statistical Analysis

The VAS score was the dependent variable, with Tool (Wheelchair, Rollerblades), Ending (Success, Safe, Fall) and Intensity of State (Negative, Neutral) as independent variables.

All the analyses were conducted following a Bayesian approach, similar to the one used for the Action Anticipation data (see below C). We computed the modes (Mo) and HPDIs for the μ parameters of the β coefficients.

The mathematical description of the Hierarchical Bayesian Linear Model is reported in Table A.1 and the corresponding JAGS code (the script for the Bayesian analysis) is reported in Code A.1.

The comparisons (contrasts) among coefficients are reported on Table A.2

Estimation of the Coefficients via Hierarchical Bayesian Linear Model with non-informative prior

Table A.1: Hierarchical Bayesian Model for the non-informative prior for the Linear Model

**Formula**	**Rows in the JAGS code**	**Description**
$y_i \sim Normal(\mu_i, \sigma^2)$	4	Likelihood
$\mu_i=\alpha+\beta_v+\beta_t+\beta_e+\beta_{v:t}+\beta_{v:e}+\beta_{t:e}+\beta_{v:t:e}+\beta_s$	6-8	Linear model
$\alpha \sim Normal(0, \sigma^2_\alpha)$	55	Prior for α
$\beta_v \sim Normal(0, \sigma^2_v)$	44	Prior for β_v
$\beta_e \sim Normal(0, \sigma^2_e)$	40	Prior for β_e
$\beta_t \sim Normal(0, \sigma^2_t)$	48	Prior for β_t
$\beta_{v:e} \sim Normal(0, \sigma^2_{v:e})$	23	Prior for $\beta_{v:e}$
$\beta_{v:t} \sim Normal(0, \sigma^2_{v:t})$	29	Prior for $\beta_{v:t}$

$\beta_{t:e}$~*Normal(0, $\sigma^2_{t:e}$)*	35	Prior for $\beta_{t:e}$
$\beta_{v:t:e}$~*Normal(0, $\sigma^2_{v:t:e}$)*	16	Prior for $\beta_{v:t:e}$
β_s~*Normal(0, σ^2_s)*	52	Prior for β_s
σ^2…~Uniform(0.001, 1000)	58-66	Hyperprior. Prior for all the σ^2…

Code A.1: JAGS code for the Hierarchical Bayesian Linear Model for the non-informative prior for the Emotional Valence of the Stimuli

```
1   model {
2   # Likelihood.
3   for(iin1:Ntotal){
4     y[i]~dnorm(mu[i] , 1/pow(sigma,2))
5
6     mu[i]<-alpha+B_v[v[i]]+B_t[t[i]]+B_e[e[i]]+
7          B_vt[v[i],t[i]]+B_ve[v[i],e[i]]+B_te[t[i],e[i]]+
8     B_vte[v[i],t[i],e[i]]+ B_s[subj[i]]
9   }
10
11    sigma ~dunif(0.001,1000)
12
13  for(iv in1:nvalence){
14    for(it in1:ntype){
15      for(iein1:nending){
16        B_vte[iv,it,ie]~dnorm(0.0 ,1/pow(VTE_Sigma,2))
17      }
18    }
19  }
20
21  for(iv in1:nvalence){
22    for(iein1:nending){
23      B_ve[iv,ie]~dnorm(0.0 ,1/pow(VE_Sigma,2))
24    }
25  }
26
27  for(iv in1:nvalence){
28    for(it in1:ntype){
29      B_vt[iv,it]~dnorm(0.0 ,1/pow(VT_Sigma,2))
30    }
31  }
32
33  for(it in1:ntype){
34    for(iein1:nending){
35      B_te[it,ie]~dnorm(0.0 ,1/pow(TE_Sigma,2))
36    }
37  }
38
39  for(iein1:nending){
40    B_e[ie]~dnorm(0.0 , 1/pow(ending_Sigma,2))
41  }
42
43  for(iv in1:nvalence){
44    B_v[iv]~dnorm(0.0 , 1/pow(valence_Sigma,2))
45  }
46
47  for(it in1:ntype){
48    B_t[it]~dnorm(0.0 , 1/pow(type_Sigma,2))
49  }
50
51  for(isubjin1:nsubj){
52    B_s[isubj]~dnorm(0.0, 1/pow(subj_Sigma,2))
53  }
54
55    alpha ~dnorm(0.0 , 1/pow(alpha_Sigma,2))
```

```
## Hyperpriors
VTE_Sigma~dunif(0.001,1000)
TE_Sigma~dunif(0.001,1000)
VT_Sigma~dunif(0.001,1000)
VE_Sigma~dunif(0.001,1000)
valence_Sigma~ dunif(0.001,1000)
ending_Sigma~ dunif(0.001,1000)
type_Sigma~ dunif(0.001,1000)
subj_Sigma~ dunif(0.001,1000)
alpha_Sigma~ dunif(0.001,1000)

## Code for Sum-to-zero coefficients
## ...
```

Table A.2: Contrasts from the posterior distributions

The direct contrasts between posterior distributions are reported in each row. A contrast is the difference between two posterior distributions. For example, the "Rollerblade v Wheelchair" contrast is the posterior distribution of the difference between "Rollerblade" and "Wheelchair".

Results are described by means of the Mode, the 89%HPDI, the ESS and the $\hat{R}$ value. Contrasts whose HPDIs are completely greater than the ROPE are marked with "+" (i.e., the first term of the contrast is larger than the second term), conversely, they are marked with "-" (i.e., the second term of the contrast is larger than the first term). These are considered credible differences/contrasts. Contrasts that are not marked with a "+" or a "-" are considered non-credible.

In the table it is observable that only contrasts within main effects and the Emotion:Ending interaction are giving credible results.

As reported in the main text, these analyses show that Fall and Safe endings elicit more negative emotions (anxiety and unpleasantness) than Success endings, while neutral emotions (arousal and unexpectedness) are elicited more by Success endings than Safe and Fall endings. In addition, Wheelchair videos were related to higher negative evaluations than Rollerblades videos.

**Main Effects Contrasts**	Mode	HPDI		ESS	$\hat{R}$	
Rollerblade v. Wheelchair	-3.199	-4.428	-1.904	120086.8	1	-
Safe Fail v. Fall	-6.963	-8.392	-5.300	125000.0	1	-
Safe Fail v. Success	5.248	3.657	6.732	122421.6	1	+
Fall v. Success	12.099	10.472	13.575	111245.7	1	+
Negative v. Neutral	-4.511	-5.804	-3.293	125000.0	1	-
**Emotion:Ending contrasts**						
Negative: Safe Fail v. Fall	-0.891	-2.414	0.594	125000.00	1	
Negative: Safe Fail v. Success	3.572	1.970	5.047	80587.34	1	+
Negative: Fall v. Success	4.349	2.872	5.998	66430.18	1	+
Neutral: Safe Fail v. Fall	0.891	-0.594	2.414	125000.00	1	
Neutral: Safe Fail v. Success	-3.572	-5.047	-1.970	80587.34	1	-
Neutral: Fall v. Success	-4.349	-5.998	-2.872	66430.18	1	-
Safe Fail: Negative v. Neutral	1.662	-0.009	3.465	118276.68	1	
Fall: Negative v. Neutral	3.569	1.754	5.281	87336.94	1	+

Success: Negative v. Neutral	-5.249	-7.134	-3.521	62725.78	1	-
**Emotion:Tool Contrasts**						
Negative: Wheelchair v. Rollerblades	1.071	-0.150	2.321	39802.35	1	
Neutral: Wheelchair v. Rollerblades	-1.071	-2.321	0.150	39802.35	1	
Wheelchair: Negative v. Neutral	1.071	-0.150	2.321	39802.35	1	
Rollerblades: Negative v. Neutral	-1.071	-2.321	0.150	39802.35	1	
**Valence:Tool Contrasts**						
Safe Fail: Wheelchair v. Rollerblades	-1.233	-2.943	0.375	22233.50	1	
Fall: Wheelchair v. Rollerblades	1.450	-0.154	3.304	16276.95	1	
Success: Wheelchair v. Rollerblades	-0.098	-1.941	1.211	95962.82	1	
Wheelchair: Safe Fail v. Fall	-1.351	-2.924	0.095	15549.53	1	
Wheelchair: Safe Fail v. Success	-0.315	-1.840	0.908	64943.57	1	
Wheelchair: Fall v. Success	0.766	-0.401	2.438	27841.38	1	
Rollerblades: Safe Fail v. Fall	1.351	-0.095	2.924	15549.53	1	
Rollerblades: Safe Fail v. Success	0.315	-0.908	1.840	64943.57	1	
Rollerblades: Fall v. Success	-0.766	-2.438	0.401	27841.38	1	
**Valence:Ending:Tool Contrasts**						
Negative X Rollerblades X Safe Fail v. Negative X Rollerblades X Fall	-0.005	-0.861	1.107	86238.59	1	
Negative X Rollerblades X Safe Fail v. Negative X Rollerblades X Success	0.001	-0.991	0.955	125000.00	1	
Negative X Rollerblades X Fall v. Negative X Rollerblades X Success	0.002	-1.107	0.867	71300.34	1	
Negative X Wheelchair X Safe Fail v. Negative X Wheelchair X Fall	0.005	-1.107	0.861	86238.59	1	
Negative X Wheelchair X Safe Fail v. Negative X Wheelchair X Success	-0.001	-0.955	0.991	125000.00	1	
Negative X Wheelchair X Fall v. Negative X Wheelchair X Success	-0.002	-0.867	1.107	71300.34	1	
Neutral X Rollerblades X Safe Fail v. Neutral X Rollerblades X Fall	0.005	-1.107	0.861	86238.59	1	
Neutral X Rollerblades X Safe Fail v. Neutral X Rollerblades X Success	-0.001	-0.955	0.991	125000.00	1	
Neutral X Rollerblades X Fall v. Neutral X Rollerblades X Success	-0.002	-0.867	1.107	71300.34	1	
Neutral X Wheelchair X Safe Fail v. Neutral X Wheelchair X Fall	-0.005	-0.861	1.107	86238.59	1	
Neutral X Wheelchair X Safe Fail v. Neutral X Wheelchair X Success	0.001	-0.991	0.955	125000.00	1	
Neutral X Wheelchair X Fall v.	0.002	-1.107	0.867	71300.34	1	

Neutral X Wheelchair X Success					
Negative X Rollerblades X Safe Fail v. Negative X Wheelchair X Safe Fail	-0.007	-1.042	1.206	116871.90	1
Negative X Rollerblades X Fall v. Negative X Wheelchair X Fall	-0.006	-1.300	0.983	77652.19	1
Negative X Rollerblades X Success v. Negative X Wheelchair X Success	0.004	-1.005	1.259	107361.80	1
Neutral X Rollerblades X Safe Fail v. Neutral X Wheelchair X Safe Fail	0.007	-1.206	1.042	116871.90	1
Neutral X Rollerblades X Fall v. Neutral X Wheelchair X Fall	0.006	-0.983	1.300	77652.19	1
Neutral X Rollerblades X Success v. Neutral X Wheelchair X Success	-0.004	-1.259	1.005	107361.80	1
Negative X Rollerblades X Safe Fail v. Neutral X Rollerblades X Safe Fail	-0.007	-1.042	1.206	116871.90	1
Negative X Wheelchair X Safe Fail v. Neutral X Wheelchair X Safe Fail	0.007	-1.206	1.042	116871.90	1
Negative X Rollerblades X Fall v. Neutral X Rollerblades X Fall	-0.006	-1.300	0.983	77652.19	1
Negative X Wheelchair X Fall v. Neutral X Wheelchair X Fall	0.006	-0.983	1.300	77652.19	1
Negative X Rollerblades X Success v. Neutral X Rollerblades X Success	0.004	-1.005	1.259	107361.80	1
Negative X Wheelchair X Success v. Neutral X Wheelchair X Success	-0.004	-1.259	1.005	107361.80	1

B. Analysis of Kinematics

In order to test the kinematic differences between the different Endings (Success, Safe Fail, Fall) and Tools (Wheelchair, Rollerblades), and whether or not the six versions of the videos for each Ending and Tool where kinematically different, we calculated the angles formed by the actors' right elbow and left knee with respect to the floor, and the distance covered by the video actor in the four earlier frames: at the starting position (0 ms), and during the actions after 600 ms, 1200 ms and 1800 ms.

We chose these information because they are representative of the whole action in both rollerblade and wheelchair videos: the right elbow angle is important for wheelchair users to push their wheelchair and for rollerblade users to maintain balance; the left knee angle in respect to the floor shows the preparation for the jumps in rollerblades, and is an indirect measure of the degree of the wheelchair tilt necessary in order to climb the platform; finally, the distance covered is an index of velocity (see Fig. B.1).

Figure B.1: Graphical representation of angles used for kinematic analyses.

In red the left knee angle with respect to the floor, in blue the right elbow angle. a = rollerblades video; b= wheelchair video.

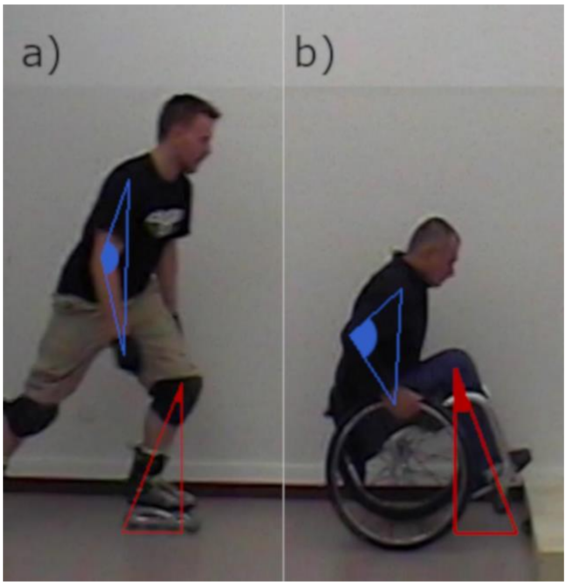

The definition of the angles and the distance covered was performed using the free and open-source software Kinovea ver. 0.8.15 (Copyright 2006-2011 Joan Charmant & Contributors – www.kinovea.org).

Data handling and Statistical Analyses

The kinematic information was obtained from the 36 videos used in the Action Anticipation paradigm (see the main text) in the frames at 0 ms, 600 ms, 1200 ms and 1800 ms.

First, for each combination of Ending and Tool, we tested if there were differences between the six videos. Three separated analyses were conducted with the distance covered (in pixels), the right elbow angle (in radians), the left knee angle (in radians) as dependent variables (d.v.). The ID of the Video and the Frame (0, 600, 1200, 1800 ms) were the independent variables.

As a second step, we tested if there were kinematic differences between Tools and Endings. We tested all the above-mentioned d.v., with Ending (Success, Safe Fail, Fall), Tool (Wheelchair, Rollerblades) and Frame (0, 600, 1200, 1800 ms) as independent variables.

All the analyses were conducted following a Bayesian approach, similar to the one used for the Action Anticipation data (see below C). We computed the modes (Mo) and HPDIs for the μ parameters of the β coefficients.

The mathematical description of the Hierarchical Bayesian Linear Model is reported in Table B.1 (differences among videos) and Table B.2 (differences among tools and endings) and the corresponding JAGS codes (the script for the Bayesian analysis) are reported in Code B.1 and Code B.2, respectively.

The coefficients reported in Table B.3 and Table B.4 are the same as seen in Table A.2.

In Table B.3 we can observe that there are no differences between the six versions of the same video, both for the main effects and the covariation with Frame (0, 600, 1200 and 1800 ms).

The contrasts concern the comparisons between the main effects of the videos, and then their covariation with the Frame.

In all cases, the different six version of videos were not different in kinematics.

In Table B.4 we report the overall analyses of video kinematics, with Frame (0, 600, 1200 and 1800 ms), Ending (Safe Fail, Success, Fall) and Tool (Wheelchair, Rollerblades) as independent variables. As described in the main text, the left knee angle is only able to discriminate between Wheelchair and Rollerblade Videos, as expected. In fact, while in Rollerblades Videos the left knee has a great modulation, the left knee in Wheelchair Videos is only able to detect the tilt of the Wheelchair, which occurs in later frames. Furthermore, the direction of the angle in Wheelchair and Rollerblades Videos is opposite (as seen in Fig. B.1).

The right elbow angle is able to discriminate between the different tools, but also between different Endings. In particular, there is a specific pattern with Success > Fall > Safe Fail. In Wheelchair Videos Safe Fail have greater angles than Success Endings, while the opposite relation is observable in Rollerblades videos. Finally, in Safe Fail Endings, Wheelchair Videos have greater angles than Rollerblades videos.

Distances are able to discriminate between Wheelchair and Rollerblades Videos, and between Success, Fall and Safe Fail Videos.

The ROPE for the angles was [-0.02; 0.02], while for distance was [-2; 2].

Estimation of the Coefficients via Hierarchical Bayesian Linear Model with non-informative prior

Table B.1: Hierarchical Bayesian Model for the non-informative prior for the Linear Model to test differences among videos

Formula	Rows in the JAGS code	Description
$y_i \sim Normal(\mu_i, \sigma^2)$	4	Likelihood
$\mu_i = \alpha + \beta_f + \beta_v + \beta_{f:v}$	5	Linear model
$\alpha \sim Normal(0, \sigma^2_\alpha)$	15	Prior for α
$\beta f \sim Normal(0, \sigma^2_f)$	13	Prior for β_f
$\beta_v \sim Normal(0, \sigma^2_v)$	9	Prior for β_v
$\beta_{f:v} \sim Normal(0, \sigma^2_{f:v})$	10	Prior for $\beta_{f:v}$
$\sigma^2 ... \sim Uniform(0.01, 100)$	17-20	Hyperprior. Prior for all the σ^2...

Code B.1: JAGS code for the Hierarchical Bayesian Linear Model for the non-informative prior to test differences among videos

```
1   model {
2   ## Likelihood
3   for(i in 1:Ntotal){
4           y[i]~dnorm(mu[i], 1/pow(sigma[i],2))
5           mu[i]<- alpha + B_v[v[i]] + frame[i]*(B_f+B_vf[v[i]])
6   }
7
8   for(iv in 1:nvideo){
9           B_v[iv]~dnorm(0.0 , 1/pow(video_Sigma,2))
10          B_vf[iv]~dnorm(0.0 , 1/pow(frame_Sigma,2))
11  }
12
13  B_f ~ dnorm(0.0 , 1/pow(length_Sigma,2))
14
15  alpha ~dnorm(0.0 , 0.001)
16
17  ## Hyperpriors
18  frame_Sigma     ~dunif(0.01,100)
19  video_Sigma     ~dunif(0.01,100)
20  sigma           ~dunif(0.01,100)
21
22  ## Code for Sum-to-zero coefficients
23  ## ...
    }
```

Table B.2: Hierarchical Bayesian Model for the non-informative prior for the Linear Model to test differences among Endings and Tools

Formula	Rows in the JAGS code	Description
$y_i \sim Normal(\mu_i, \sigma^2)$	4	Likelihood
$\mu_i = \alpha + \beta_f + \beta_e + \beta_t + \beta_{t:e} + \beta_{f:e} + \beta_{t:f} + \beta_{t:e:f}$	6-7	Linear model
$\alpha \sim Normal(0, \sigma^2_\alpha)$	29	Prior for α
$\beta_f \sim Normal(0, \sigma^2_f)$	27	Prior for β_f

β_e~*Normal(0, $\sigma^2{}_e$)*	18	Prior for β_e
β_t~*Normal(0, $\sigma^2{}_e$)*	23	Prior for β_t
$\beta_{e:f}$~*Normal(0, $\sigma^2{}_{e:f}$)*	19	Prior for $\beta_{e:f}$
$B_{t:e}$~*Normal(0, $\sigma^2{}_{t:e}$)*	12	Prior for $\beta_{t:e}$
$B_{t:f}$~*Normal(0, $\sigma^2{}_{t:f}$)*	24	Prior for $\beta_{t:f}$
$B_{t:e:f}$~*Normal(0, $\sigma^2{}_{t:e}$)*	13	Prior for $\beta_{t:e:f}$
σ^2*…~Uniform(0.001, 1000)*	32-36	Hyperprior. Prior for all the σ^2…

Code B.2: JAGS code for the Hierarchical Bayesian Linear Model for the non-informative prior to test differences among Endings and Tools

```
1    model {
2    ## Likelihood
3    for(i in 1:Ntotal){
4            y[i]~dnorm(mu[i], 1/pow(sigma[i],2))
5
6            mu[i]<- alpha +B_e[e[i]]+ B_t[t[i]]+B_te[t[i],e[i]]+
7                            frame[i]*(B_f+B_ef[e[i]]+B_tef[t[i],e[i]]+ B_tf[t[i]])
8    }
9
10   for(it in 1:ntool){
11           for(ie in 1:nending){
12                   B_te[it,ie] ~dnorm(0.0 , 1/pow(TE_Sigma,2))
13                   B_tef[it,ie]~dnorm(0.0 , 1/pow(frame_Sigma,2))
14           }
15   }
16
17   for(ie in 1:nending){
18           B_e[ie] ~dnorm(0.0 , 1/pow(ending_Sigma,2))
19           B_ef[ie]~dnorm(0.0 , 1/pow(frame_Sigma,2))
20   }
21
22   for(it in1:ntool){
23           B_t[it] ~dnorm(0.0 , 1/pow(tool_Sigma,2))
24           B_tf[it]~dnorm(0.0 , 1/pow(frame_Sigma,2))
25   }
26
27   B_f             ~dnorm(0.0 , 1/pow(frame_Sigma,2))
28
29   alpha           ~dnorm(0.0 , 0.001)
30
31   ## Hyperpriors
32   TE_Sigma        ~dunif(0.01,100)
33   frame_Sigma     ~dunif(0.01,100)
34   ending_Sigma    ~dunif(0.01,100)
35   tool_Sigma      ~dunif(0.01,100)
36   sigma           ~dunif(0.01,100)
37
38   ## Code for Sum-to-zero coefficients
39   ## ...
40   }
```

Table B.3: Contrasts from the posterior distributions – differences among single videos

Left Knee Angle; Video: Wheelchair; Ending: Fail

Video contrasts	Mode	HPDI		ESS	$\hat{R}$
Video 1 v. Video 2	-0.01	-0.51	0.13	16583.15	1.00
Video 1 v. Video 3	0.00	-0.35	0.22	71681.48	1.00
Video 1 v. Video 4	-0.01	-0.48	0.14	18727.91	1.00
Video 1 v. Video 5	-0.01	-0.38	0.20	38813.61	1.00
Video 1 v. Video 6	-0.01	-0.37	0.21	49549.26	1.00
Video 2 v. Video 3	0.01	-0.18	0.42	25111.42	1.00
Video 2 v. Video 4	0.00	-0.26	0.29	107262.59	1.00
Video 2 v. Video 5	0.00	-0.20	0.38	38604.48	1.00
Video 2 v. Video 6	0.01	-0.18	0.40	32265.40	1.00
Video 3 v. Video 4	0.00	-0.40	0.18	31747.11	1.00
Video 3 v. Video 5	0.00	-0.32	0.24	90502.65	1.00
Video 3 v. Video 6	0.00	-0.30	0.26	106481.12	1.00
Video 4 v. Video 5	0.00	-0.22	0.35	53438.99	1.00
Video 4 v. Video 6	0.00	-0.20	0.38	41029.77	1.00
Video 5 v. Video 6	0.00	-0.27	0.29	115356.47	1.00
**Covariation with frame contrasts**	Mode	HPDI		ESS	$\hat{R}$
Video 1 v. Video 2	0.00	0.00	0.00	32274.12	1.00
Video 1 v. Video 3	0.00	0.00	0.00	93886.13	1.00
Video 1 v. Video 4	0.00	0.00	0.00	37262.95	1.00
Video 1 v. Video 5	0.00	0.00	0.00	70209.23	1.00
Video 1 v. Video 6	0.00	0.00	0.00	84558.64	1.00
Video 2 v. Video 3	0.00	0.00	0.00	48248.82	1.00
Video 2 v. Video 4	0.00	0.00	0.00	116493.18	1.00
Video 2 v. Video 5	0.00	0.00	0.00	67744.49	1.00
Video 2 v. Video 6	0.00	0.00	0.00	57287.37	1.00
Video 3 v. Video 4	0.00	0.00	0.00	56098.73	1.00
Video 3 v. Video 5	0.00	0.00	0.00	102871.87	1.00
Video 3 v. Video 6	0.00	0.00	0.00	114014.01	1.00
Video 4 v. Video 5	0.00	0.00	0.00	82464.63	1.00
Video 4 v. Video 6	0.00	0.00	0.00	65626.39	1.00
Video 5 v. Video 6	0.00	0.00	0.00	120000.00	1.00

Right Elbow Angle; Video: Wheelchair; Ending: Fail

Video contrasts	Mode	HPDI		ESS	$\hat{R}$
Video 1 v. Video 2	0.00	-0.48	0.39	117967.88	1.00
Video 1 v. Video 3	-0.02	-1.02	0.09	12350.84	1.00
Video 1 v. Video 4	0.00	-0.47	0.40	117185.55	1.00
Video 1 v. Video 5	0.00	-0.47	0.41	117280.85	1.00
Video 1 v. Video 6	0.01	-0.35	0.52	83043.99	1.00
Video 2 v. Video 3	-0.02	-0.97	0.11	13202.30	1.00
Video 2 v. Video 4	0.00	-0.43	0.45	120000.00	1.00
Video 2 v. Video 5	0.00	-0.45	0.43	121176.97	1.00
Video 2 v. Video 6	0.00	-0.32	0.56	67263.58	1.00
Video 3 v. Video 4	0.03	-0.10	0.99	13722.91	1.00
Video 3 v. Video 5	0.02	-0.11	0.96	13678.05	1.00
Video 3 v. Video 6	0.02	-0.07	1.12	10833.02	1.00
Video 4 v. Video 5	0.00	-0.45	0.43	120000.00	1.00

	Mode	HPDI		ESS	$\hat{R}$
Video 4 v. Video 6	0.01	-0.33	0.55	70158.94	1.00
Video 5 v. Video 6	0.01	-0.30	0.59	63216.33	1.00
**Covariation with frame contrasts**	Mode	HPDI		ESS	$\hat{R}$
Video 1 v. Video 2	0.00	0.00	0.00	120000.00	1.00
Video 1 v. Video 3	0.00	0.00	0.00	19001.44	1.00
Video 1 v. Video 4	0.00	0.00	0.00	120000.00	1.00
Video 1 v. Video 5	0.00	0.00	0.00	119313.98	1.00
Video 1 v. Video 6	0.00	0.00	0.00	102254.90	1.00
Video 2 v. Video 3	0.00	0.00	0.00	20568.25	1.00
Video 2 v. Video 4	0.00	0.00	0.00	120000.00	1.00
Video 2 v. Video 5	0.00	0.00	0.00	120000.00	1.00
Video 2 v. Video 6	0.00	0.00	0.00	88479.68	1.00
Video 3 v. Video 4	0.00	0.00	0.00	21073.69	1.00
Video 3 v. Video 5	0.00	0.00	0.00	21120.45	1.00
Video 3 v. Video 6	0.00	0.00	0.00	15768.71	1.00
Video 4 v. Video 5	0.00	0.00	0.00	121578.94	1.00
Video 4 v. Video 6	0.00	0.00	0.00	89618.49	1.00
Video 5 v. Video 6	0.00	0.00	0.00	89302.95	1.00

Distance; Video: Wheelchair; Ending: Fail

Video contrasts	Mode	HPDI		ESS	$\hat{R}$
Video 1 v. Video 2	0.12	-18.06	25.65	46924.00	1.00
Video 1 v. Video 3	0.28	-14.54	30.95	20454.33	1.00
Video 1 v. Video 4	-0.06	-13.81	31.98	18920.94	1.00
Video 1 v. Video 5	-0.06	-14.02	31.53	18699.27	1.00
Video 1 v. Video 6	0.18	-10.38	39.73	9680.96	1.00
Video 2 v. Video 3	-0.14	-17.76	25.82	56540.26	1.00
Video 2 v. Video 4	-0.11	-18.09	25.69	50555.63	1.00
Video 2 v. Video 5	-0.01	-17.65	25.74	50135.18	1.00
Video 2 v. Video 6	0.25	-12.94	33.58	15125.78	1.00
Video 3 v. Video 4	-0.11	-20.83	22.41	120000.00	1.00
Video 3 v. Video 5	-0.12	-20.69	22.47	120000.00	1.00
Video 3 v. Video 6	0.07	-15.53	29.01	29552.32	1.00
Video 4 v. Video 5	0.09	-21.45	21.57	128258.34	1.00
Video 4 v. Video 6	-0.10	-15.55	28.87	33741.36	1.00
Video 5 v. Video 6	0.01	-15.41	28.88	31768.75	1.00
**Covariation with frame contrasts**	Mode	HPDI		ESS	$\hat{R}$
Video 1 v. Video 2	0.02	-0.01	0.04	81068.95	1.00
Video 1 v. Video 3	0.00	-0.03	0.03	46023.47	1.00
Video 1 v. Video 4	0.00	-0.03	0.03	41504.13	1.00
Video 1 v. Video 5	0.03	0.00	0.05	41725.20	1.00
Video 1 v. Video 6	0.02	-0.01	0.05	19336.95	1.00
Video 2 v. Video 3	-0.02	-0.04	0.01	86715.60	1.00
Video 2 v. Video 4	-0.01	-0.04	0.01	84602.48	1.00
Video 2 v. Video 5	0.01	-0.01	0.04	90931.34	1.00
Video 2 v. Video 6	0.00	-0.03	0.03	32486.02	1.00
Video 3 v. Video 4	0.00	-0.02	0.03	120364.51	1.00
Video 3 v. Video 5	0.03	0.00	0.05	117904.89	1.00

Video 3 v. Video 6	0.02	-0.01	0.04	58200.56	1.00
Video 4 v. Video 5	0.03	0.00	0.05	120000.00	1.00
Video 4 v. Video 6	0.02	-0.01	0.04	63903.61	1.00
Video 5 v. Video 6	-0.01	-0.04	0.02	59093.91	1.00

Left Knee Angle; Video: Wheelchair; Ending: Fall

**Video contrasts**	Mode	HPDI		ESS	$\hat{R}$
Video 1 v. Video 2	0.00	-0.06	0.11	58547.48	1.00
Video 1 v. Video 3	0.01	-0.06	0.11	57736.04	1.00
Video 1 v. Video 4	0.01	-0.04	0.14	25364.00	1.00
Video 1 v. Video 5	0.00	-0.07	0.09	95402.58	1.00
Video 1 v. Video 6	0.01	-0.06	0.11	65885.84	1.00
Video 2 v. Video 3	0.00	-0.08	0.09	120000.00	1.00
Video 2 v. Video 4	0.01	-0.06	0.12	51573.53	1.00
Video 2 v. Video 5	0.00	-0.10	0.07	87401.74	1.00
Video 2 v. Video 6	0.00	-0.09	0.08	120000.00	1.00
Video 3 v. Video 4	0.01	-0.06	0.11	52466.68	1.00
Video 3 v. Video 5	0.00	-0.10	0.07	87853.93	1.00
Video 3 v. Video 6	0.00	-0.08	0.08	118811.93	1.00
Video 4 v. Video 5	-0.01	-0.13	0.05	32773.91	1.00
Video 4 v. Video 6	-0.01	-0.12	0.06	48593.30	1.00
Video 5 v. Video 6	0.00	-0.07	0.10	100124.19	1.00
**Covariation with frame contrasts**	Mode	HPDI		ESS	$\hat{R}$
Video 1 v. Video 2	0.00	0.00	0.00	87695.80	1.00
Video 1 v. Video 3	0.00	0.00	0.00	83897.36	1.00
Video 1 v. Video 4	0.00	0.00	0.00	45074.52	1.00
Video 1 v. Video 5	0.00	0.00	0.00	114630.67	1.00
Video 1 v. Video 6	0.00	0.00	0.00	92063.65	1.00
Video 2 v. Video 3	0.00	0.00	0.00	120000.00	1.00
Video 2 v. Video 4	0.00	0.00	0.00	82031.76	1.00
Video 2 v. Video 5	0.00	0.00	0.00	106489.60	1.00
Video 2 v. Video 6	0.00	0.00	0.00	120000.00	1.00
Video 3 v. Video 4	0.00	0.00	0.00	82781.75	1.00
Video 3 v. Video 5	0.00	0.00	0.00	103358.60	1.00
Video 3 v. Video 6	0.00	0.00	0.00	118986.51	1.00
Video 4 v. Video 5	0.00	0.00	0.00	57628.76	1.00
Video 4 v. Video 6	0.00	0.00	0.00	76389.68	1.00
Video 5 v. Video 6	0.00	0.00	0.00	111622.23	1.00

Right Elbow Angle; Video: Wheelchair; Ending: Fall

**Video contrasts**	Mode	HPDI		ESS	$\hat{R}$
Video 1 v. Video 2	0.00	-0.29	0.14	34885.23	1.00
Video 1 v. Video 3	0.00	-0.23	0.19	115147.10	1.00
Video 1 v. Video 4	-0.01	-0.36	0.10	18231.71	1.00
Video 1 v. Video 5	0.00	-0.25	0.16	60999.18	1.00
Video 1 v. Video 6	0.00	-0.25	0.16	63671.95	1.00
Video 2 v. Video 3	0.00	-0.15	0.27	45855.36	1.00
Video 2 v. Video 4	0.00	-0.26	0.16	52122.22	1.00

Video 2 v. Video 5	0.00	-0.18	0.23	94199.99	1.00
Video 2 v. Video 6	0.00	-0.17	0.24	84505.01	1.00
Video 3 v. Video 4	-0.01	-0.33	0.11	21675.63	1.00
Video 3 v. Video 5	0.00	-0.23	0.18	90252.41	1.00
Video 3 v. Video 6	0.00	-0.23	0.18	88761.16	1.00
Video 4 v. Video 5	0.01	-0.14	0.29	31303.03	1.00
Video 4 v. Video 6	0.01	-0.13	0.30	30810.25	1.00
Video 5 v. Video 6	0.00	-0.20	0.20	121553.70	1.00

**Covariation with frame contrasts**	Mode	HPDI		ESS	$\hat{R}$
Video 1 v. Video 2	0.00	0.00	0.00	64928.79	1.00
Video 1 v. Video 3	0.00	0.00	0.00	120000.00	1.00
Video 1 v. Video 4	0.00	0.00	0.00	36779.00	1.00
Video 1 v. Video 5	0.00	0.00	0.00	94579.00	1.00
Video 1 v. Video 6	0.00	0.00	0.00	94556.08	1.00
Video 2 v. Video 3	0.00	0.00	0.00	79048.22	1.00
Video 2 v. Video 4	0.00	0.00	0.00	85243.63	1.00
Video 2 v. Video 5	0.00	0.00	0.00	114711.74	1.00
Video 2 v. Video 6	0.00	0.00	0.00	105437.82	1.00
Video 3 v. Video 4	0.00	0.00	0.00	40013.43	1.00
Video 3 v. Video 5	0.00	0.00	0.00	111975.04	1.00
Video 3 v. Video 6	0.00	0.00	0.00	106640.23	1.00
Video 4 v. Video 5	0.00	0.00	0.00	60420.97	1.00
Video 4 v. Video 6	0.00	0.00	0.00	57390.12	1.00
Video 5 v. Video 6	0.00	0.00	0.00	120543.85	1.00

Distance; Video: Wheelchair; Ending: Fall

**Video contrasts**	Mode	HPDI		ESS	$\hat{R}$
Video 1 v. Video 2	0.08	-32.96	18.82	45406.46	1.00
Video 1 v. Video 3	0.03	-27.79	23.06	96415.26	1.00
Video 1 v. Video 4	-0.33	-44.30	12.72	13862.45	1.00
Video 1 v. Video 5	0.16	-25.80	25.11	118050.73	1.00
Video 1 v. Video 6	0.25	-25.81	24.82	118886.99	1.00
Video 2 v. Video 3	0.08	-21.31	30.06	73603.43	1.00
Video 2 v. Video 4	-0.27	-36.41	16.81	25963.10	1.00
Video 2 v. Video 5	-0.09	-19.83	31.88	50930.65	1.00
Video 2 v. Video 6	0.08	-19.35	32.63	40797.18	1.00
Video 3 v. Video 4	-0.02	-41.25	14.02	16309.98	1.00
Video 3 v. Video 5	0.04	-23.96	26.88	114788.59	1.00
Video 3 v. Video 6	0.09	-22.59	28.46	97349.71	1.00
Video 4 v. Video 5	0.30	-13.49	42.79	14385.37	1.00
Video 4 v. Video 6	0.56	-13.39	43.96	13227.11	1.00
Video 5 v. Video 6	0.00	-23.86	26.86	115491.58	1.00
**Covariation with frame contrasts**	Mode	HPDI		ESS	$\hat{R}$
Video 1 v. Video 2	-0.04	-0.07	-0.01	76211.72	1.00
Video 1 v. Video 3	0.00	-0.03	0.03	115832.89	1.00
Video 1 v. Video 4	-0.05	-0.08	-0.02	29249.29	1.00
Video 1 v. Video 5	0.03	0.00	0.06	118312.39	1.00
Video 1 v. Video 6	0.01	-0.02	0.04	120096.37	1.00

Video 2 v. Video 3	0.05	0.01	0.07	104916.37	1.00
Video 2 v. Video 4	-0.01	-0.04	0.03	55228.72	1.00
Video 2 v. Video 5	0.07	0.04	0.10	84860.73	1.00
Video 2 v. Video 6	0.06	0.03	0.09	73221.74	1.00
Video 3 v. Video 4	-0.05	-0.08	-0.02	33496.69	1.00
Video 3 v. Video 5	0.03	0.00	0.06	118394.75	1.00
Video 3 v. Video 6	0.02	-0.02	0.04	114116.48	1.00
Video 4 v. Video 5	0.08	0.04	0.11	31318.58	1.00
Video 4 v. Video 6	0.06	0.03	0.09	27185.10	1.00
Video 5 v. Video 6	-0.01	-0.04	0.02	118940.83	1.00

Left Knee Angle; Video: Wheelchair; Ending: Success

**Video contrasts**	Mode	HPDI		ESS	$\hat{R}$
Video 1 v. Video 2	0.00	-0.14	0.15	120376.56	1.00
Video 1 v. Video 3	-0.01	-0.21	0.10	30453.17	1.00
Video 1 v. Video 4	0.00	-0.18	0.11	57445.75	1.00
Video 1 v. Video 5	0.00	-0.16	0.13	103408.68	1.00
Video 1 v. Video 6	0.00	-0.17	0.13	78512.66	1.00
Video 2 v. Video 3	-0.01	-0.21	0.10	32854.47	1.00
Video 2 v. Video 4	-0.01	-0.18	0.11	61493.80	1.00
Video 2 v. Video 5	0.00	-0.15	0.14	109017.19	1.00
Video 2 v. Video 6	0.00	-0.16	0.13	84453.47	1.00
Video 3 v. Video 4	0.00	-0.12	0.17	72540.81	1.00
Video 3 v. Video 5	0.00	-0.11	0.19	44131.59	1.00
Video 3 v. Video 6	0.00	-0.11	0.18	58910.63	1.00
Video 4 v. Video 5	0.00	-0.12	0.17	86867.58	1.00
Video 4 v. Video 6	0.00	-0.13	0.16	108718.81	1.00
Video 5 v. Video 6	0.00	-0.16	0.13	114409.42	1.00
**Covariation with frame contrasts**	Mode	HPDI		ESS	$\hat{R}$
Video 1 v. Video 2	0.00	0.00	0.00	121873.44	1.00
Video 1 v. Video 3	0.00	0.00	0.00	58501.78	1.00
Video 1 v. Video 4	0.00	0.00	0.00	89580.47	1.00
Video 1 v. Video 5	0.00	0.00	0.00	118852.55	1.00
Video 1 v. Video 6	0.00	0.00	0.00	103006.00	1.00
Video 2 v. Video 3	0.00	0.00	0.00	61699.97	1.00
Video 2 v. Video 4	0.00	0.00	0.00	99865.18	1.00
Video 2 v. Video 5	0.00	0.00	0.00	116216.99	1.00
Video 2 v. Video 6	0.00	0.00	0.00	111407.98	1.00
Video 3 v. Video 4	0.00	0.00	0.00	95555.01	1.00
Video 3 v. Video 5	0.00	0.00	0.00	74245.80	1.00
Video 3 v. Video 6	0.00	0.00	0.00	92199.22	1.00
Video 4 v. Video 5	0.00	0.00	0.00	105542.38	1.00
Video 4 v. Video 6	0.00	0.00	0.00	120000.00	1.00
Video 5 v. Video 6	0.00	0.00	0.00	120000.00	1.00

Right Elbow Angle; Video: Wheelchair; Ending: Success

**Video contrasts**	Mode	HPDI		ESS	$\hat{R}$
Video 1 v. Video 2	-0.01	-0.25	0.14	40622.14	1.00

Video 1 v. Video 3	0.00	-0.17	0.21	98764.51	1.00
Video 1 v. Video 4	0.00	-0.24	0.14	47697.76	1.00
Video 1 v. Video 5	0.00	-0.24	0.14	51893.18	1.00
Video 1 v. Video 6	0.00	-0.24	0.14	51914.66	1.00
Video 2 v. Video 3	0.01	-0.12	0.27	30434.75	1.00
Video 2 v. Video 4	0.00	-0.18	0.19	125916.74	1.00
Video 2 v. Video 5	0.00	-0.18	0.19	111413.31	1.00
Video 2 v. Video 6	0.00	-0.18	0.19	119011.26	1.00
Video 3 v. Video 4	0.00	-0.27	0.13	33729.69	1.00
Video 3 v. Video 5	0.00	-0.26	0.13	36150.41	1.00
Video 3 v. Video 6	0.00	-0.27	0.12	34029.76	1.00
Video 4 v. Video 5	0.00	-0.18	0.20	119462.62	1.00
Video 4 v. Video 6	0.00	-0.19	0.19	123527.41	1.00
Video 5 v. Video 6	0.00	-0.18	0.19	118892.99	1.00
**Covariation with frame contrasts**	Mode	HPDI		ESS	$\hat{R}$
Video 1 v. Video 2	0.00	0.00	0.00	80812.70	1.00
Video 1 v. Video 3	0.00	0.00	0.00	110690.46	1.00
Video 1 v. Video 4	0.00	0.00	0.00	84784.90	1.00
Video 1 v. Video 5	0.00	0.00	0.00	90342.95	1.00
Video 1 v. Video 6	0.00	0.00	0.00	88306.80	1.00
Video 2 v. Video 3	0.00	0.00	0.00	59739.89	1.00
Video 2 v. Video 4	0.00	0.00	0.00	120000.00	1.00
Video 2 v. Video 5	0.00	0.00	0.00	118833.26	1.00
Video 2 v. Video 6	0.00	0.00	0.00	121209.68	1.00
Video 3 v. Video 4	0.00	0.00	0.00	63843.54	1.00
Video 3 v. Video 5	0.00	0.00	0.00	65782.19	1.00
Video 3 v. Video 6	0.00	0.00	0.00	64694.52	1.00
Video 4 v. Video 5	0.00	0.00	0.00	120000.00	1.00
Video 4 v. Video 6	0.00	0.00	0.00	121218.80	1.00
Video 5 v. Video 6	0.00	0.00	0.00	120000.00	1.00

Distance; Video: Wheelchair; Ending: Success

**Video contrasts**	Mode	HPDI		ESS	$\hat{R}$
Video 1 v. Video 2	-0.38	-74.27	59.01	118806.37	1.00
Video 1 v. Video 3	-89.75	-162.83	3.41	14717.31	1.00
Video 1 v. Video 4	-1.72	-73.69	60.29	120000.00	1.00
Video 1 v. Video 5	0.84	-62.16	72.20	115573.99	1.00
Video 1 v. Video 6	-0.53	-69.22	64.00	120000.00	1.00
Video 2 v. Video 3	-84.42	-155.95	5.83	16915.32	1.00
Video 2 v. Video 4	0.41	-67.81	66.05	120000.00	1.00
Video 2 v. Video 5	1.47	-57.65	76.46	106473.88	1.00
Video 2 v. Video 6	0.57	-63.37	70.31	118426.16	1.00
Video 3 v. Video 4	84.44	-5.06	154.72	17186.75	1.00
Video 3 v. Video 5	102.14	-3.33	166.69	13602.57	1.00
Video 3 v. Video 6	85.78	-3.98	159.14	16222.13	1.00
Video 4 v. Video 5	1.40	-55.65	79.10	103094.46	1.00
Video 4 v. Video 6	0.23	-64.49	69.91	120000.00	1.00
Video 5 v. Video 6	-0.94	-74.82	59.28	117095.45	1.00

Covariation with frame contrasts	Mode	HPDI		ESS	$\hat{R}$
Video 1 v. Video 2	-0.01	-0.07	0.05	117436.36	1.00
Video 1 v. Video 3	0.06	-0.02	0.13	23992.84	1.00
Video 1 v. Video 4	0.01	-0.05	0.07	118923.79	1.00
Video 1 v. Video 5	-0.01	-0.07	0.06	116395.33	1.00
Video 1 v. Video 6	0.00	-0.06	0.06	120000.00	1.00
Video 2 v. Video 3	0.07	-0.01	0.14	24838.24	1.00
Video 2 v. Video 4	0.02	-0.04	0.08	118019.36	1.00
Video 2 v. Video 5	0.00	-0.06	0.07	116912.11	1.00
Video 2 v. Video 6	0.01	-0.05	0.07	120000.00	1.00
Video 3 v. Video 4	-0.05	-0.11	0.03	26494.60	1.00
Video 3 v. Video 5	-0.07	-0.14	0.01	21353.09	1.00
Video 3 v. Video 6	-0.06	-0.13	0.02	25080.01	1.00
Video 4 v. Video 5	-0.02	-0.08	0.04	108433.91	1.00
Video 4 v. Video 6	-0.01	-0.08	0.05	120000.00	1.00
Video 5 v. Video 6	0.01	-0.05	0.07	116891.27	1.00

Left Knee Angle; Video: Rollerblades; Ending: Fail

Video contrasts	Mode	HPDI		ESS	$\hat{R}$
Video 1 v. Video 2	0.00	-0.10	0.10	117726.51	1.00
Video 1 v. Video 3	0.01	-0.06	0.15	35038.60	1.00
Video 1 v. Video 4	0.00	-0.09	0.10	118707.32	1.00
Video 1 v. Video 5	0.00	-0.09	0.10	113011.65	1.00
Video 1 v. Video 6	0.01	-0.06	0.15	31529.20	1.00
Video 2 v. Video 3	0.01	-0.06	0.15	32408.03	1.00
Video 2 v. Video 4	0.00	-0.09	0.11	120000.00	1.00
Video 2 v. Video 5	0.00	-0.09	0.11	111996.73	1.00
Video 2 v. Video 6	0.01	-0.06	0.16	29766.98	1.00
Video 3 v. Video 4	-0.01	-0.15	0.06	39389.86	1.00
Video 3 v. Video 5	-0.01	-0.15	0.06	42252.87	1.00
Video 3 v. Video 6	0.00	-0.10	0.10	117059.57	1.00
Video 4 v. Video 5	0.00	-0.10	0.10	120000.00	1.00
Video 4 v. Video 6	0.01	-0.06	0.15	35192.05	1.00
Video 5 v. Video 6	0.01	-0.06	0.15	38776.43	1.00
**Covariation with frame contrasts**	Mode	HPDI		ESS	$\hat{R}$
Video 1 v. Video 2	0.00	0.00	0.00	120000.00	1.00
Video 1 v. Video 3	0.00	0.00	0.00	62003.06	1.00
Video 1 v. Video 4	0.00	0.00	0.00	120000.00	1.00
Video 1 v. Video 5	0.00	0.00	0.00	120000.00	1.00
Video 1 v. Video 6	0.00	0.00	0.00	53840.82	1.00
Video 2 v. Video 3	0.00	0.00	0.00	57698.77	1.00
Video 2 v. Video 4	0.00	0.00	0.00	120000.00	1.00
Video 2 v. Video 5	0.00	0.00	0.00	118844.34	1.00
Video 2 v. Video 6	0.00	0.00	0.00	51636.31	1.00
Video 3 v. Video 4	0.00	0.00	0.00	66221.28	1.00
Video 3 v. Video 5	0.00	0.00	0.00	69807.84	1.00
Video 3 v. Video 6	0.00	0.00	0.00	120000.00	1.00
Video 4 v. Video 5	0.00	0.00	0.00	120000.00	1.00

Video 4 v. Video 6	0.00	0.00	0.00	57908.93	1.00
Video 5 v. Video 6	0.00	0.00	0.00	62004.94	1.00

Right Elbow Angle; Video: Rollerblades; Ending: Fail

**Video contrasts**	Mode	HPDI		ESS	$\hat{R}$
Video 1 v. Video 2	0.00	-0.47	0.45	116923.05	1.00
Video 1 v. Video 3	0.00	-0.43	0.48	114258.05	1.00
Video 1 v. Video 4	0.00	-0.40	0.53	94044.67	1.00
Video 1 v. Video 5	-0.01	-0.84	0.21	16253.81	1.00
Video 1 v. Video 6	0.00	-0.47	0.45	114806.14	1.00
Video 2 v. Video 3	0.00	-0.42	0.50	111857.15	1.00
Video 2 v. Video 4	0.00	-0.41	0.52	90498.73	1.00
Video 2 v. Video 5	-0.01	-0.82	0.21	16218.05	1.00
Video 2 v. Video 6	0.00	-0.48	0.45	120000.00	1.00
Video 3 v. Video 4	0.00	-0.43	0.49	115758.33	1.00
Video 3 v. Video 5	-0.02	-0.87	0.20	15122.19	1.00
Video 3 v. Video 6	0.00	-0.50	0.42	102920.04	1.00
Video 4 v. Video 5	-0.02	-0.92	0.19	13115.60	1.00
Video 4 v. Video 6	0.00	-0.54	0.39	75116.75	1.00
Video 5 v. Video 6	0.01	-0.22	0.82	18016.81	1.00
**Covariation with frame contrasts**	Mode	HPDI		ESS	$\hat{R}$
Video 1 v. Video 2	0.00	0.00	0.00	120600.42	1.00
Video 1 v. Video 3	0.00	0.00	0.00	120000.00	1.00
Video 1 v. Video 4	0.00	0.00	0.00	112571.21	1.00
Video 1 v. Video 5	0.00	0.00	0.00	29231.30	1.00
Video 1 v. Video 6	0.00	0.00	0.00	120000.00	1.00
Video 2 v. Video 3	0.00	0.00	0.00	117357.61	1.00
Video 2 v. Video 4	0.00	0.00	0.00	108872.91	1.00
Video 2 v. Video 5	0.00	0.00	0.00	28075.14	1.00
Video 2 v. Video 6	0.00	0.00	0.00	120000.00	1.00
Video 3 v. Video 4	0.00	0.00	0.00	115065.47	1.00
Video 3 v. Video 5	0.00	0.00	0.00	27579.74	1.00
Video 3 v. Video 6	0.00	0.00	0.00	114286.41	1.00
Video 4 v. Video 5	0.00	0.00	0.00	24851.16	1.00
Video 4 v. Video 6	0.00	0.00	0.00	105870.20	1.00
Video 5 v. Video 6	0.00	0.00	0.00	32880.44	1.00

Distance; Video: Rollerblades; Ending: Fail

**Video contrasts**	Mode	HPDI		ESS	$\hat{R}$
Video 1 v. Video 2	0.04	-30.58	35.69	103324.09	1.00
Video 1 v. Video 3	-0.02	-36.01	29.97	99239.07	1.00
Video 1 v. Video 4	-0.07	-30.57	35.60	102090.95	1.00
Video 1 v. Video 5	-0.01	-32.03	34.11	109217.79	1.00
Video 1 v. Video 6	0.33	-27.84	38.69	64836.05	1.00
Video 2 v. Video 3	-0.27	-37.87	28.90	64968.13	1.00
Video 2 v. Video 4	-0.36	-32.88	33.10	121154.80	1.00
Video 2 v. Video 5	-0.33	-34.19	31.85	120000.00	1.00
Video 2 v. Video 6	0.23	-30.97	35.17	100222.52	1.00

	Mode	HPDI		ESS	$\hat{R}$
Video 3 v. Video 4	-0.14	-28.10	38.37	64788.21	1.00
Video 3 v. Video 5	0.11	-28.72	37.51	71148.85	1.00
Video 3 v. Video 6	0.04	-26.25	41.58	41150.30	1.00
Video 4 v. Video 5	0.31	-33.33	32.68	113674.47	1.00
Video 4 v. Video 6	0.35	-29.42	36.72	98579.77	1.00
Video 5 v. Video 6	0.17	-29.94	36.42	84868.81	1.00
**Covariation with frame contrasts**	Mode	HPDI		ESS	$\hat{R}$
Video 1 v. Video 2	0.02	-0.03	0.06	111117.42	1.00
Video 1 v. Video 3	-0.02	-0.07	0.02	114009.23	1.00
Video 1 v. Video 4	-0.01	-0.06	0.04	120000.00	1.00
Video 1 v. Video 5	-0.02	-0.06	0.03	120000.00	1.00
Video 1 v. Video 6	0.00	-0.04	0.05	103576.15	1.00
Video 2 v. Video 3	-0.04	-0.08	0.01	88765.82	1.00
Video 2 v. Video 4	-0.03	-0.07	0.02	120000.00	1.00
Video 2 v. Video 5	-0.03	-0.08	0.01	118508.10	1.00
Video 2 v. Video 6	-0.01	-0.06	0.03	117375.70	1.00
Video 3 v. Video 4	0.01	-0.03	0.06	95122.12	1.00
Video 3 v. Video 5	0.01	-0.04	0.05	112566.71	1.00
Video 3 v. Video 6	0.03	-0.02	0.07	77740.72	1.00
Video 4 v. Video 5	-0.01	-0.05	0.04	120000.00	1.00
Video 4 v. Video 6	0.01	-0.03	0.06	120000.00	1.00
Video 5 v. Video 6	0.02	-0.03	0.06	117023.26	1.00

Left Knee Angle; Video: Rollerblades; Ending: Fall

**Video contrasts**	Mode	HPDI		ESS	$\hat{R}$
Video 1 v. Video 2	-0.01	-0.10	0.06	71172.22	1.00
Video 1 v. Video 3	-0.01	-0.10	0.06	79206.55	1.00
Video 1 v. Video 4	0.01	-0.06	0.09	92268.84	1.00
Video 1 v. Video 5	-0.01	-0.13	0.04	36819.36	1.00
Video 1 v. Video 6	-0.01	-0.14	0.04	29011.14	1.00
Video 2 v. Video 3	0.00	-0.07	0.08	120000.00	1.00
Video 2 v. Video 4	0.01	-0.05	0.12	46133.98	1.00
Video 2 v. Video 5	-0.01	-0.10	0.06	71852.38	1.00
Video 2 v. Video 6	-0.01	-0.11	0.05	52116.16	1.00
Video 3 v. Video 4	0.01	-0.05	0.11	50289.96	1.00
Video 3 v. Video 5	-0.01	-0.10	0.06	65989.54	1.00
Video 3 v. Video 6	-0.01	-0.11	0.05	46647.63	1.00
Video 4 v. Video 5	-0.02	-0.14	0.03	26067.60	1.00
Video 4 v. Video 6	-0.02	-0.15	0.03	22426.29	1.00
Video 5 v. Video 6	0.00	-0.09	0.07	108224.49	1.00
**Covariation with frame contrasts**	Mode	HPDI		ESS	$\hat{R}$
Video 1 v. Video 2	0.00	0.00	0.00	91248.18	1.00
Video 1 v. Video 3	0.00	0.00	0.00	101754.04	1.00
Video 1 v. Video 4	0.00	0.00	0.00	106667.66	1.00
Video 1 v. Video 5	0.00	0.00	0.00	54141.16	1.00
Video 1 v. Video 6	0.00	0.00	0.00	47757.29	1.00
Video 2 v. Video 3	0.00	0.00	0.00	120375.09	1.00
Video 2 v. Video 4	0.00	0.00	0.00	70040.21	1.00

Video 2 v. Video 5	0.00	0.00	0.00	94386.97	1.00
Video 2 v. Video 6	0.00	0.00	0.00	76892.54	1.00
Video 3 v. Video 4	0.00	0.00	0.00	76279.69	1.00
Video 3 v. Video 5	0.00	0.00	0.00	91881.35	1.00
Video 3 v. Video 6	0.00	0.00	0.00	72387.21	1.00
Video 4 v. Video 5	0.00	0.00	0.00	40723.48	1.00
Video 4 v. Video 6	0.00	0.00	0.00	35941.08	1.00
Video 5 v. Video 6	0.00	0.00	0.00	115562.34	1.00

Right Elbow Angle; Video: Rollerblades; Ending: Fall

Video contrasts	Mode	HPDI		ESS	$\hat{R}$
Video 1 v. Video 2	0.01	-0.31	0.92	19371.15	1.00
Video 1 v. Video 3	0.01	-0.28	1.00	15430.21	1.00
Video 1 v. Video 4	0.00	-0.40	0.78	31427.11	1.00
Video 1 v. Video 5	0.00	-0.53	0.61	99700.48	1.00
Video 1 v. Video 6	0.01	-0.49	0.66	73994.98	1.00
Video 2 v. Video 3	0.01	-0.48	0.65	98344.05	1.00
Video 2 v. Video 4	0.00	-0.67	0.48	76393.97	1.00
Video 2 v. Video 5	0.00	-0.82	0.38	26650.39	1.00
Video 2 v. Video 6	-0.01	-0.80	0.38	32116.52	1.00
Video 3 v. Video 4	-0.01	-0.76	0.41	41333.17	1.00
Video 3 v. Video 5	-0.01	-0.91	0.32	18260.75	1.00
Video 3 v. Video 6	-0.01	-0.89	0.33	21660.96	1.00
Video 4 v. Video 5	0.00	-0.71	0.45	49948.94	1.00
Video 4 v. Video 6	-0.01	-0.69	0.46	62671.32	1.00
Video 5 v. Video 6	0.00	-0.54	0.60	120000.00	1.00
**Covariation with frame contrasts**	Mode	HPDI		ESS	$\hat{R}$
Video 1 v. Video 2	0.00	0.00	0.00	39879.62	1.00
Video 1 v. Video 3	0.00	0.00	0.00	30570.68	1.00
Video 1 v. Video 4	0.00	0.00	0.00	58781.98	1.00
Video 1 v. Video 5	0.00	0.00	0.00	116367.96	1.00
Video 1 v. Video 6	0.00	0.00	0.00	100594.06	1.00
Video 2 v. Video 3	0.00	0.00	0.00	115048.90	1.00
Video 2 v. Video 4	0.00	0.00	0.00	102449.21	1.00
Video 2 v. Video 5	0.00	0.00	0.00	52075.58	1.00
Video 2 v. Video 6	0.00	0.00	0.00	58074.27	1.00
Video 3 v. Video 4	0.00	0.00	0.00	71670.08	1.00
Video 3 v. Video 5	0.00	0.00	0.00	37847.77	1.00
Video 3 v. Video 6	0.00	0.00	0.00	41745.69	1.00
Video 4 v. Video 5	0.00	0.00	0.00	85254.99	1.00
Video 4 v. Video 6	0.00	0.00	0.00	96271.52	1.00
Video 5 v. Video 6	0.00	0.00	0.00	118917.21	1.00

Distance; Video: Rollerblades; Ending: Fall

Video contrasts	Mode	HPDI		ESS	$\hat{R}$
Video 1 v. Video 2	0.03	-44.16	31.79	60968.79	1.00
Video 1 v. Video 3	0.02	-39.46	36.51	120000.00	1.00
Video 1 v. Video 4	-0.13	-46.36	30.88	52212.81	1.00

Video 1 v. Video 5	-0.38	-39.81	35.85	113026.06	1.00
Video 1 v. Video 6	-0.15	-43.00	32.55	82896.31	1.00
Video 2 v. Video 3	-0.07	-32.39	42.86	80231.61	1.00
Video 2 v. Video 4	0.30	-39.11	36.51	120000.00	1.00
Video 2 v. Video 5	0.33	-33.71	42.04	89373.78	1.00
Video 2 v. Video 6	-0.06	-36.58	39.03	111319.18	1.00
Video 3 v. Video 4	0.27	-43.90	32.49	64674.53	1.00
Video 3 v. Video 5	-0.09	-37.90	37.65	119468.54	1.00
Video 3 v. Video 6	-0.42	-39.86	36.01	96116.05	1.00
Video 4 v. Video 5	-0.24	-32.10	44.06	63161.53	1.00
Video 4 v. Video 6	-0.24	-33.34	42.53	99893.19	1.00
Video 5 v. Video 6	0.27	-40.97	34.70	109365.00	1.00
**Covariation with frame contrasts**	Mode	HPDI		ESS	$\hat{R}$
Video 1 v. Video 2	-0.03	-0.08	0.02	97252.17	1.00
Video 1 v. Video 3	-0.03	-0.08	0.02	115722.20	1.00
Video 1 v. Video 4	-0.03	-0.08	0.02	92800.82	1.00
Video 1 v. Video 5	-0.01	-0.07	0.04	118210.37	1.00
Video 1 v. Video 6	-0.01	-0.06	0.04	118390.87	1.00
Video 2 v. Video 3	0.00	-0.05	0.05	116970.55	1.00
Video 2 v. Video 4	0.00	-0.05	0.05	120000.00	1.00
Video 2 v. Video 5	0.01	-0.04	0.07	109761.72	1.00
Video 2 v. Video 6	0.01	-0.04	0.07	120000.00	1.00
Video 3 v. Video 4	0.00	-0.05	0.05	111945.00	1.00
Video 3 v. Video 5	0.01	-0.04	0.06	118778.27	1.00
Video 3 v. Video 6	0.01	-0.04	0.06	120000.00	1.00
Video 4 v. Video 5	0.02	-0.04	0.07	105618.27	1.00
Video 4 v. Video 6	0.02	-0.03	0.07	120000.00	1.00
Video 5 v. Video 6	0.00	-0.05	0.05	118871.22	1.00

Left Knee Angle; Video: Rollerblades; Ending: Success

**Video contrasts**	Mode	HPDI		ESS	$\hat{R}$
Video 1 v. Video 2	0.00	-0.11	0.16	90134.48	1.00
Video 1 v. Video 3	-0.01	-0.18	0.10	59286.34	1.00
Video 1 v. Video 4	-0.01	-0.24	0.06	20462.54	1.00
Video 1 v. Video 5	-0.01	-0.18	0.09	51803.33	1.00
Video 1 v. Video 6	-0.01	-0.22	0.08	28756.72	1.00
Video 2 v. Video 3	-0.01	-0.20	0.08	35927.72	1.00
Video 2 v. Video 4	-0.02	-0.27	0.05	16004.41	1.00
Video 2 v. Video 5	-0.01	-0.21	0.08	33680.43	1.00
Video 2 v. Video 6	-0.01	-0.24	0.06	20110.98	1.00
Video 3 v. Video 4	-0.01	-0.20	0.09	36167.53	1.00
Video 3 v. Video 5	0.00	-0.14	0.13	121266.08	1.00
Video 3 v. Video 6	-0.01	-0.17	0.10	65280.01	1.00
Video 4 v. Video 5	0.01	-0.09	0.19	40302.33	1.00
Video 4 v. Video 6	0.00	-0.11	0.16	84610.16	1.00
Video 5 v. Video 6	0.00	-0.16	0.11	73307.21	1.00
**Covariation with frame contrasts**	Mode	HPDI		ESS	$\hat{R}$
Video 1 v. Video 2	0.00	0.00	0.00	111531.98	1.00

Video 1 v. Video 3	0.00	0.00	0.00	88450.63	1.00
Video 1 v. Video 4	0.00	0.00	0.00	36808.45	1.00
Video 1 v. Video 5	0.00	0.00	0.00	83033.70	1.00
Video 1 v. Video 6	0.00	0.00	0.00	50226.39	1.00
Video 2 v. Video 3	0.00	0.00	0.00	58704.16	1.00
Video 2 v. Video 4	0.00	0.00	0.00	27566.42	1.00
Video 2 v. Video 5	0.00	0.00	0.00	53525.39	1.00
Video 2 v. Video 6	0.00	0.00	0.00	35063.52	1.00
Video 3 v. Video 4	0.00	0.00	0.00	64108.07	1.00
Video 3 v. Video 5	0.00	0.00	0.00	121092.82	1.00
Video 3 v. Video 6	0.00	0.00	0.00	93158.38	1.00
Video 4 v. Video 5	0.00	0.00	0.00	67300.46	1.00
Video 4 v. Video 6	0.00	0.00	0.00	108349.28	1.00
Video 5 v. Video 6	0.00	0.00	0.00	103231.07	1.00

Right Elbow Angle; Video: Rollerblades; Ending: Success

**Video contrasts**	Mode	HPDI		ESS	$\hat{R}$
Video 1 v. Video 2	0.00	-0.27	0.42	65306.55	1.00
Video 1 v. Video 3	0.02	-0.13	0.70	13218.27	1.00
Video 1 v. Video 4	0.01	-0.19	0.56	21278.35	1.00
Video 1 v. Video 5	0.00	-0.31	0.38	105087.72	1.00
Video 1 v. Video 6	0.00	-0.24	0.48	43078.48	1.00
Video 2 v. Video 3	0.01	-0.18	0.59	18261.44	1.00
Video 2 v. Video 4	0.01	-0.25	0.46	41450.30	1.00
Video 2 v. Video 5	0.00	-0.39	0.30	97541.27	1.00
Video 2 v. Video 6	0.00	-0.31	0.39	100419.33	1.00
Video 3 v. Video 4	-0.01	-0.45	0.25	48970.65	1.00
Video 3 v. Video 5	-0.01	-0.65	0.15	14261.78	1.00
Video 3 v. Video 6	-0.01	-0.55	0.19	23454.75	1.00
Video 4 v. Video 5	-0.01	-0.53	0.21	26640.75	1.00
Video 4 v. Video 6	-0.01	-0.43	0.27	66593.26	1.00
Video 5 v. Video 6	0.00	-0.27	0.44	63389.05	1.00
**Covariation with frame contrasts**	Mode	HPDI		ESS	$\hat{R}$
Video 1 v. Video 2	0.00	0.00	0.00	94284.30	1.00
Video 1 v. Video 3	0.00	0.00	0.00	24560.40	1.00
Video 1 v. Video 4	0.00	0.00	0.00	41756.15	1.00
Video 1 v. Video 5	0.00	0.00	0.00	114470.84	1.00
Video 1 v. Video 6	0.00	0.00	0.00	73062.31	1.00
Video 2 v. Video 3	0.00	0.00	0.00	34885.97	1.00
Video 2 v. Video 4	0.00	0.00	0.00	70684.85	1.00
Video 2 v. Video 5	0.00	0.00	0.00	114613.22	1.00
Video 2 v. Video 6	0.00	0.00	0.00	116848.58	1.00
Video 3 v. Video 4	0.00	0.00	0.00	81275.64	1.00
Video 3 v. Video 5	0.00	0.00	0.00	25433.17	1.00
Video 3 v. Video 6	0.00	0.00	0.00	43359.60	1.00
Video 4 v. Video 5	0.00	0.00	0.00	48929.97	1.00
Video 4 v. Video 6	0.00	0.00	0.00	91167.24	1.00
Video 5 v. Video 6	0.00	0.00	0.00	92816.49	1.00

Distance; Video: Rollerblades; Ending: Success					
**Video contrasts**	Mode	HPDI		ESS	$\hat{R}$
Video 1 v. Video 2	0.01	-37.89	25.93	52350.29	1.00
Video 1 v. Video 3	-0.01	-32.02	31.44	110624.32	1.00
Video 1 v. Video 4	-0.09	-33.94	29.33	108100.92	1.00
Video 1 v. Video 5	-0.20	-40.69	23.75	37087.68	1.00
Video 1 v. Video 6	-0.11	-34.35	28.95	100457.31	1.00
Video 2 v. Video 3	-0.10	-27.30	36.62	65917.80	1.00
Video 2 v. Video 4	-0.04	-28.71	34.63	87236.65	1.00
Video 2 v. Video 5	0.18	-34.72	28.42	113828.80	1.00
Video 2 v. Video 6	-0.14	-27.71	35.73	77145.95	1.00
Video 3 v. Video 4	-0.29	-32.60	30.67	118006.02	1.00
Video 3 v. Video 5	0.10	-37.95	26.16	49645.30	1.00
Video 3 v. Video 6	0.33	-33.23	30.10	110979.87	1.00
Video 4 v. Video 5	0.18	-36.28	27.53	58624.60	1.00
Video 4 v. Video 6	0.04	-31.99	31.35	122410.59	1.00
Video 5 v. Video 6	-0.12	-27.13	36.84	56108.72	1.00
**Covariation with frame contrasts**	Mode	HPDI		ESS	$\hat{R}$
Video 1 v. Video 2	0.00	-0.04	0.04	88226.28	1.00
Video 1 v. Video 3	0.03	-0.01	0.08	109133.55	1.00
Video 1 v. Video 4	0.00	-0.04	0.04	117669.85	1.00
Video 1 v. Video 5	-0.02	-0.06	0.03	83310.31	1.00
Video 1 v. Video 6	0.00	-0.04	0.05	116509.06	1.00
Video 2 v. Video 3	0.04	-0.01	0.08	92681.53	1.00
Video 2 v. Video 4	0.00	-0.04	0.05	107667.00	1.00
Video 2 v. Video 5	-0.01	-0.05	0.03	118325.74	1.00
Video 2 v. Video 6	0.01	-0.04	0.05	112448.19	1.00
Video 3 v. Video 4	-0.03	-0.07	0.01	114376.39	1.00
Video 3 v. Video 5	-0.05	-0.09	0.00	79968.20	1.00
Video 3 v. Video 6	-0.03	-0.07	0.01	113631.27	1.00
Video 4 v. Video 5	-0.02	-0.06	0.03	98763.13	1.00
Video 4 v. Video 6	0.00	-0.04	0.04	121269.21	1.00
Video 5 v. Video 6	0.02	-0.02	0.06	99405.89	1.00

Table B.4: Contrasts from the posterior distributions – differences among Tools and Endings

Left Knee Angle	Mode	HPDI		ESS	$\hat{R}$
Wheelchair v. Rollerblades	-0.29	-0.36	-0.21	116248.11	1.00 -
Safe Fail v. Fall	0.02	-0.05	0.11	90501.57	1.00
Safe Fail v. Success	0.02	-0.05	0.11	80550.98	1.00
Fall v. Success	0.00	-0.08	0.08	116182.06	1.00
Wheelchair: Safe Fail v. Fall	0.00	-0.05	0.07	67321.40	1.00
Wheelchair: Safe Fail v. Success	0.00	-0.05	0.07	64528.81	1.00

Wheelchair: Fall v. Success	0.00	-0.06	0.06	120000.00	1.00
Rollerblades: Safe Fail v. Fall	0.00	-0.07	0.05	67321.40	1.00
Rollerblades: Safe Fail v. Success	0.00	-0.07	0.05	64528.81	1.00
Rollerblades: Fall v. Success	0.00	-0.06	0.06	120000.00	1.00
Safe Fail: Wheelchair v. Rollerblades	0.01	-0.05	0.09	57465.41	1.00
Fall: Wheelchair v. Rollerblades	0.00	-0.08	0.06	94989.92	1.00
Success: Wheelchair v. Rollerblades	0.00	-0.08	0.06	97966.86	1.00
Covariation with Frame - Safe Fail v. Fall	0.00	-0.02	0.02	123379.88	1.00
Covariation with Frame - Safe Fail v. Success	0.00	-0.02	0.02	120000.00	1.00
Covariation with Frame - Fall v. Success	0.00	-0.02	0.02	120000.00	1.00
Covariation with Frame - Wheelchair: Safe Fail v. Fall	0.00	-0.02	0.02	123401.02	1.00
Covariation with Frame - Wheelchair: Safe Fail v. Success	0.00	-0.02	0.02	120000.00	1.00
Covariation with Frame - Wheelchair: Fall v. Success	0.00	-0.02	0.02	120000.00	1.00
Covariation with Frame - Rollerblades: Safe Fail v. Fall	0.00	-0.02	0.02	123390.99	1.00
Covariation with Frame - Rollerblades: Safe Fail v. Success	0.00	-0.02	0.02	120000.00	1.00
Covariation with Frame - Rollerblades: Fall v. Success	0.00	-0.02	0.02	120000.00	1.00
Covariation with Frame - Safe Fail: Wheelchair v. Rollerblades	0.00	-0.01	0.01	120000.00	1.00
Covariation with Frame - Fall: Wheelchair v. Rollerblades	0.00	-0.01	0.01	120000.00	1.00
Covariation with Frame - Success: Wheelchair v. Rollerblades	0.00	-0.01	0.01	120000.00	1.00

**Right Elbow Angle**	Mode	HPDI		ESS	$\hat{R}$	
Wheelchair v. Rollerblades	-0.27	-0.43	-0.13	104118.81	1.00	-
Safe Fail v. Fall	-0.20	-0.38	-0.02	103071.15	1.00	-
Safe Fail v. Success	-0.40	-0.58	-0.22	69583.13	1.00	-
Fall v. Success	-0.20	-0.38	-0.02	102203.24	1.00	-
Wheelchair: Safe Fail v. Fall	0.16	-0.02	0.33	30906.44	1.00	
Wheelchair: Safe Fail v. Success	0.25	0.04	0.42	18064.12	1.00	+
Wheelchair: Fall v. Success	0.08	-0.08	0.24	72250.87	1.00	
Rollerblades: Safe Fail v. Fall	-0.16	-0.33	0.02	30906.44	1.00	
Rollerblades: Safe Fail v. Success	-0.25	-0.42	-0.04	18064.12	1.00	-
Rollerblades: Fall v. Success	-0.08	-0.24	0.08	72250.87	1.00	
Safe Fail: Wheelchairv. Rollerblades	0.26	0.04	0.48	19496.42	1.00	+
Fall: Wheelchairv. Rollerblades	-0.03	-0.24	0.14	104581.18	1.00	
Success: Wheelchairv. Rollerblades	-0.20	-0.41	0.00	25499.04	1.00	
Covariation with Frame - Safe Fail v. Fall	0.00	-0.02	0.02	120000.00	1.00	

Covariation with Frame - Safe Fail v. Success	0.00	-0.02	0.02	120000.00	1.00	
Covariation with Frame - Fall v. Success	0.00	-0.02	0.02	120000.00	1.00	
Covariation with Frame - Wheelchair: Safe Fail v. Fall	0.00	-0.02	0.02	120000.00	1.00	
Covariation with Frame - Wheelchair: Safe Fail v. Success	0.00	-0.02	0.02	120000.00	1.00	
Covariation with Frame - Wheelchair: Fall v. Success	0.00	-0.02	0.02	120000.00	1.00	
Covariation with Frame - Rollerblades: Safe Fail v. Fall	0.00	-0.02	0.02	120000.00	1.00	
Covariation with Frame - Rollerblades: Safe Fail v. Success	0.00	-0.02	0.02	120000.00	1.00	
Covariation with Frame - Rollerblades: Fall v. Success	0.00	-0.02	0.02	120000.00	1.00	
Covariation with Frame - Safe Fail: Wheelchair v. Rollerblades	0.00	-0.01	0.01	121190.43	1.00	
Covariation with Frame - Fall: Wheelchair v. Rollerblades	0.00	-0.01	0.01	121188.11	1.00	
Covariation with Frame - Success: Wheelchair v. Rollerblades	0.00	-0.01	0.01	121176.84	1.00	

**Distance**	Mode	HPDI		ESS	$\hat{R}$	
Wheelchair v. Rollerblades	30.50	14.73	49.23	81404.18	1.00	+
Safe Fail v. Fall	8.72	-11.43	28.26	102473.94	1.00	
Safe Fail v. Success	-22.90	-43.68	-2.41	58232.14	1.00	-
Fall v. Success	-31.50	-52.43	-9.70	43604.88	1.00	-
Wheelchair: Safe Fail v. Fall	0.18	-10.12	22.17	40866.43	1.00	
Wheelchair: Safe Fail v. Success	-0.32	-25.18	8.03	21393.95	1.00	
Wheelchair: Fall v. Success	-0.41	-31.29	4.44	10719.02	1.00	
Rollerblades: Safe Fail v. Fall	-0.18	-22.17	10.12	40866.43	1.00	
Rollerblades: Safe Fail v. Success	0.32	-8.03	25.18	21393.95	1.00	
Rollerblades: Fall v. Success	0.41	-4.44	31.29	10719.02	1.00	
Safe Fail: Wheelchairv. Rollerblades	0.03	-20.44	16.55	104240.62	1.00	
Fall: Wheelchairv. Rollerblades	-0.50	-32.25	7.24	14372.63	1.00	
Success: Wheelchairv. Rollerblades	0.44	-5.76	34.85	11752.04	1.00	
Covariation with Frame - Safe Fail v. Fall	0.01	-0.06	0.08	120000.00	1.00	
Covariation with Frame - Safe Fail v. Success	0.03	-0.04	0.10	120000.00	1.00	
Covariation with Frame - Fall v. Success	0.02	-0.05	0.09	120460.57	1.00	
Covariation with Frame - Wheelchair: Safe Fail v. Fall	0.00	-0.07	0.07	120000.00	1.00	
Covariation with Frame - Wheelchair: Safe Fail v. Success	-0.02	-0.09	0.06	115543.57	1.00	
Covariation with Frame - Wheelchair: Fall v. Success	-0.02	-0.09	0.06	95069.69	1.00	
Covariation with Frame - Rollerblades: Safe Fail v. Fall	0.01	-0.06	0.09	120000.00	1.00	
Covariation with Frame - Rollerblades: Safe Fail v. Success	0.04	-0.03	0.12	116502.96	1.00	

Covariation with Frame - Rollerblades: Fall v. Success	0.03	-0.04	0.10	100529.88	1.00
Covariation with Frame - Safe Fail: Wheelchair v. Rollerblades	-0.03	-0.09	0.04	116061.98	1.00
Covariation with Frame - Fall: Wheelchair v. Rollerblades	-0.02	-0.08	0.05	100399.15	1.00
Covariation with Frame - Success: Wheelchair v. Rollerblades	0.03	-0.03	0.10	86269.22	1.00

C. Action Anticipation Model

Figure C.1: Goodness of fit

The figure shows a graphical representation of the posterior distributions resulting from the Hierarchical Bayesian Logit models (parts A and C) and theactual data of the experiment (parts B and D), for a comparison. The similarities between posterior distribution and actual data (A v. B and C v. D) indicate that the model perfectly fits with the data.

The posterior distribution values were transformed by means of the inverse logit function (Λ^{-1}) in order to have a direct comparison with the actual data. We usedboxplots for the representation of the actual data. The darker line in the middle of the box indicates the median, the boundaries of the box represent the firstand the third quartile and the bottom and top whiskers represent the first quarter minus 1.5 *Interquartile Range and the third quarter plus 1.5 * Interquartile Range, respectively. The Points are data outside the whiskers. For representations of the posterior distribution we chose violin plots. The darker line in the middle of the box is the median, and the upper and lower boundaries of the box represent the firstand the third quartile. The curves are probability density curves represented along the y-axis instead of the x-axis, plotted on each side.

A = posterior distributions of 600-1200ms data; B = actual 600-1200 ms data; C = posterior distribution of 2400-3000ms data; D = actual 2400-3000 ms data.

A) 600-1200 ms – Posterior distribution

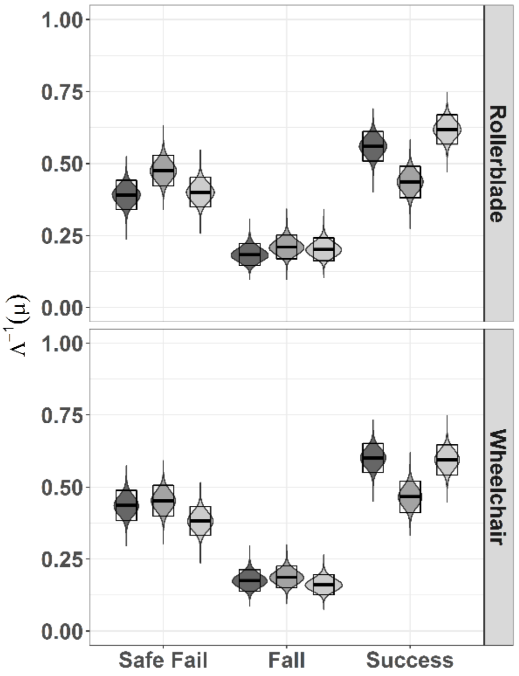

B) 600 – 1800 ms – Actual Data

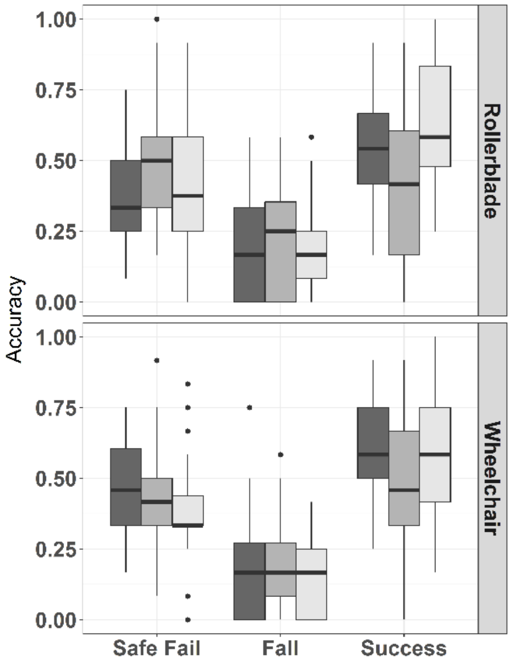

C) 2400 – 3000 ms – Posterior distribution

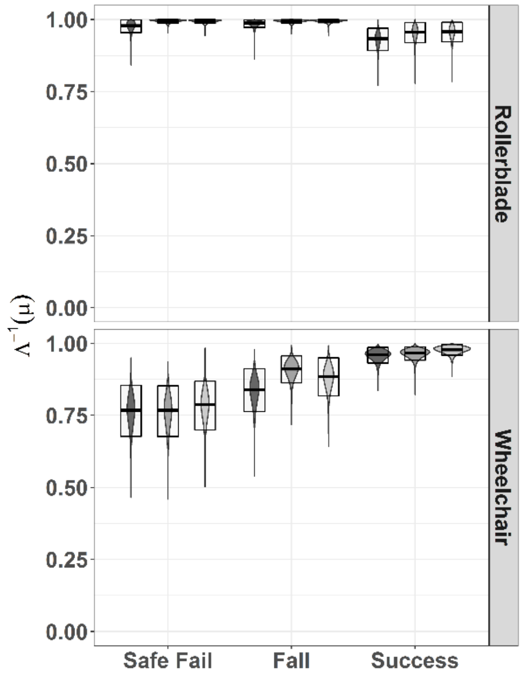

D) 2400 – 3000 ms – Actual Data

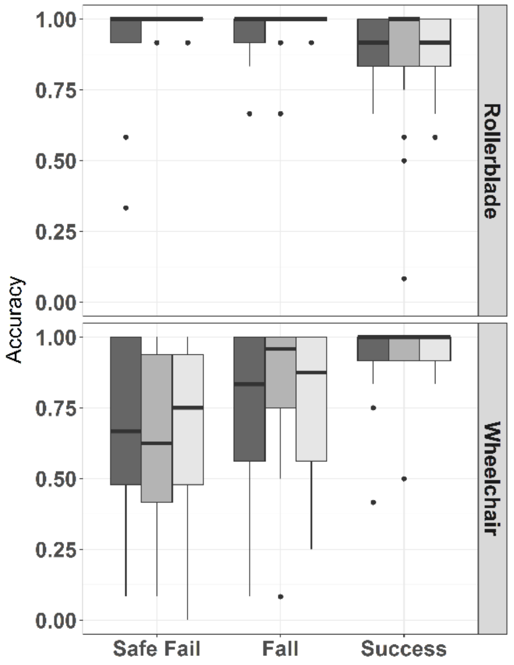

Estimation of the Coefficients via Hierarchical Bayesian Logit Model with non-informative prior

The estimation of coefficients is done through a standard Hierarchical Bayesian Logit Model (Gelman & Hill, 2006; Kruschke, 2011, 2014). This model allows us to relate binomial dependent variables (accuracies, frequencies, etc...) to linear coefficients.This is possible by assuming a normal latent variable)(an unobserved continuous variable underlying the non-parametric dependent variable, Agresti, 2012, p. 4). Therefore, it is possible to estimate coefficients for the independent variables from a conventional linear regression and transform itsresult (the normally distributed latent variables) into a binomial dependent variable (the data) via a "link-function", that in this case is the Logit function.

The Logit function in its canonical form is $Logit(x) = \frac{1}{1+e^{-x}}$.

The inverse of the Logit function transforms the [0÷1] probability of getting correct responses into a continuous variable, that is the latent variable, which is then used to estimate the coefficients βs.

As to the Bayesian Logit Model, we are interested in the posterior distributions of the μ parameter of the β coefficients, i.e. the parameter of central tendency for each independent variable. These posterior distributions are then used to compute the contrasts of interest that are reported in the paper in terms of Mode and 89% HPDI.

The mathematical description of the Hierarchical Bayesian Logit Model is reported in Table A.1, and the corresponding JAGS code (the script for the Bayesian analysis) is reported in Code A.1.

Table C.1: Hierarchical Bayesian Logit Model for the Binomial data

In the first column the formula for each term of the model. In the second column, the corresponding row in the JAGS code (see below). In the third column a short description for each term.

y_i is the dependent variable (i.e. the number of correct responses in the i^{th}case).

N_i is the number of trials in the ith case.

$length_i$ is the covariate in the ith case: the duration of the video centred and re-scaled in the [-1; 1] range.

Subscripts of βs: G = "Group" (ROL, PHY, SKA); E = "Ending" (Fall, Safe fail, Success); T = "Tool" (Rollerblade, Wheelchair); L = "Length" (a covariate from 600 to 1800ms in the first application of the model, from 2400 to 3000ms in the second one. In both cases it was centred and re-scaled in the [-1; 1] range); S = "Subject".

Formula	Rows in the JAGS code	Description
$y_i \sim Binomial(\mu_i, N_i)$	4	Likelihood
$\mu_i = Logit[\alpha + \beta_G + \beta_E + \beta_T + \beta_{G:E} + \beta_{G:T} + \beta_{E:T} + \beta_{G:E:T} + length_i \times (\beta_L + \beta_{G:L} + \beta_{E:L} + \beta_{T:L} + \beta_{G:E:L} + \beta_{G:T:L} + \beta_{E:T:L} + \beta_{G:E:T:L}) + \beta_S]$	6-12	Logit-linear model
$\alpha \sim Normal(0,0.001)$	69	Prior for α
$\beta_G \sim Normal(0,\sigma^2_G)$	59	Prior for β_G
$\beta_E \sim Normal(0,\sigma^2_E)$	49	Prior for β_E
$\beta_T \sim Normal(0,\sigma^2_T)$	54	Prior for β_T
$\beta_L \sim Normal(0,\sigma^2_{G:L})$	67	Prior for β_L
$\beta_{G:E} \sim Normal(0,\sigma^2_{G:E})$	36	Prior for $\beta_{G:E}$
$\beta_{G:T} \sim Normal(0,\sigma^2_{G:T})$	43	Prior for $\beta_{G:T}$
$\beta_{G:L} \sim Normal(0,\sigma^2_{G:L})$	60	Prior for $\beta_{G:L}$
$B_{E:L} \sim Normal(0,\sigma^2_{E:L})$	50	Prior for $\beta_{E:L}$
$\beta_{E:T} \sim Normal(0,\sigma^2_{E:T})$	29	Prior for $\beta_{E:T}$
$\beta_{T:L} \sim Normal(0,\sigma^2_{T:L})$	55	Prior for $\beta_{T:L}$
$\beta_{G:E:L} \sim Normal(0,\sigma^2_{G:E:L})$	37	Prior for $\beta_{G:E:L}$
$\beta_{G:E:T} \sim Normal(0,\sigma^2_{G:E:T})$	21	Prior for $\beta_{G:E:T}$
$\beta_{G:T:L} \sim Normal(0,\sigma^2_{G:T:L})$	44	Prior for $\beta_{G:T:L}$
$\beta_{E:T:L} \sim Normal(0,\sigma^2_{E:T:L})$	30	Prior for $\beta_{E:T:L}$
$\beta_{G:E:T:L} \sim Normal(0,\sigma^2_{G:E:T:L})$	22	Prior for $\beta_{G:E:T:L}$
$\beta_S \sim Normal(0,\sigma^2_S)$	64	Prior for β_S
$\sigma^2_{...} \sim Uniform(0.01,100)$	81-89	Hyperpriors. Priors for all the $\sigma^2_{...}$

Code C.1: JAGS code for the Hierarchical Bayesian Model for the non-informative prior for the Logit Models

The JAGS code concerning the Bayesian model whose mathematical description is in Table A is reported below.

```
1   model {
2   ## Likelihood
3   for(iin1:Ntotal){
4           y[i]~dbin(mu[i], N[i])
5
6           logit(mu[i])<-alpha+B_g[g[i]]+B_e[e[i]]+
7                   B_t[t[i]]+B_te[t[i],e[i]]+B_gt[g[i],t[i]]+
8                   B_ge[g[i],e[i]]+B_gte[g[i],t[i],e[i]]+
9                   length[i]*(B_l+B_gl[g[i]]+B_tel[t[i],e[i]]+
10                  B_gtl[g[i],t[i]]+B_gel[g[i],e[i]]+
11                  B_el[e[i]]+B_tl[t[i]]+B_gtel[g[i],t[i],e[i]])+
12                  B_s[subj[i]])
13
14
15  }
16
17  ## Priors
```

```
for(igin1:ngroup){
        for(it in1:ntool){
                for(iein1:nending){
                        B_gte[ig,it,ie]~dnorm(0.0 ,1/pow(GTE_Sigma,2))
                        B_gtel[ig,it,ie]~dnorm(0.0 ,1/pow(length_Sigma,2))
                }
        }
}

for(it in1:ntool){
        for(iein1:nending){
                B_te[it,ie]~dnorm(0.0 , 1/pow(TE_Sigma,2))
                B_tel[it,ie]~dnorm(0.0 , 1/pow(length_Sigma,2))
        }
}

for(igin1:ngroup){
        for(iein1:nending){
                B_ge[ig,ie]~dnorm(0.0 , 1/pow(GE_Sigma,2))
                B_gel[ig,ie]~dnorm(0.0 , 1/pow(length_Sigma,2))
        }
}

for(igin1:ngroup){
        for(it in1:ntool){
                B_gt[ig,it]~dnorm(0.0 , 1/pow(GT_Sigma,2))
                B_gtl[ig,it]~dnorm(0.0 , 1/pow(length_Sigma,2))
        }
}

for(iein1:nending){
        B_e[ie]~dnorm(0.0 , 1/pow(ending_Sigma,2))
        B_el[ie]~dnorm(0.0 , 1/pow(length_Sigma,2))
}

for(it in1:ntool){
        B_t[it]~dnorm(0.0 , 1/pow(tool_Sigma,2))
        B_tl[it]~dnorm(0.0 , 1/pow(length_Sigma,2))
}

for(igin1:ngroup){
        B_g[ig]~dnorm(0.0 , 1/pow(group_Sigma,2))
        B_gl[ig]~dnorm(0.0 , 1/pow(length_Sigma,2))
}

for(isubjin1:nsubj){
        B_s[isubj]~dnorm(0.0, 1/pow(subj_Sigma,2))
}

B_l~dnorm(0.0 , 1/pow(length_Sigma,2))

alpha ~dnorm(0.0 , 0.001)

## Hyperpriors
GTE_Sigma~dunif(0.01,100)
TE_Sigma~dunif(0.01,100)
GE_Sigma~dunif(0.01,100)
GT_Sigma~dunif(0.01,100)
length_Sigma~ dunif(0.01,100)
ending_Sigma~ dunif(0.01,100)
tool_Sigma~ dunif(0.01,100)
group_Sigma~ dunif(0.01,100)
subj_Sigma~ dunif(0.01,100)

## Code for Sum-to-zero coefficients
## ...
}
```

D. Posterior distributions

In this section we report the mode, 89% HPDI, ESS and $\hat{R}$ for the coefficients from all the posterior distributions.

Table D.1 – Posterior distributions for the categorial factors for the Accuracies data for the 600-1800 ms Action Anticipation experiment

	Mode	HPDI		ESS	$\hat{R}$
Intercept	-0.533	-0.601	-0.465	109351.171	1
Group: SCI	0.030	-0.061	0.126	109414.826	1
Group: PHY	-0.039	-0.134	0.053	110000.560	1
Group: SKA	0.010	-0.084	0.104	109233.251	1
Video type: Rollerblade	0.025	-0.016	0.065	81439.493	1
Video type: Wheelchair	-0.025	-0.065	0.016	81439.493	1
Video Ending: Safe Fail	0.208	0.156	0.266	97084.871	1
Video Ending: Fall	-0.944	-1.005	-0.880	70731.674	1
Video Ending: Success	0.733	0.676	0.787	98015.794	1
SCI X Rollerblade	-0.082	-0.141	-0.027	32855.421	1
PHY X Rollerblade	0.012	-0.040	0.065	97353.001	1
SKA X Rollerblade	0.071	0.015	0.127	36629.740	1
SCI X Wheelchair	0.082	0.027	0.141	32855.421	1
PHY X Wheelchair	-0.012	-0.065	0.040	97353.001	1
SKA X Wheelchair	-0.071	-0.127	-0.015	36629.740	1
SCI X Safe Fail	-0.074	-0.149	0.003	100927.145	1
PHY X Safe Fail	0.220	0.142	0.293	84789.323	1
SKA X Safe Fail	-0.144	-0.222	-0.070	93941.015	1
SCI X Fall	-0.039	-0.130	0.040	76231.641	1
PHY X Fall	0.133	0.047	0.216	68647.554	1
SKA X Fall	-0.092	-0.174	-0.003	72767.517	1
SCI X Success	0.118	0.039	0.190	98287.586	1
PHY X Success	-0.358	-0.429	-0.272	53677.644	1
SKA X Success	0.235	0.157	0.312	70679.723	1
Rollerblade X Safe Fail	-0.027	-0.081	0.024	69336.105	1
Wheelchair X Safe Fail	0.027	-0.024	0.081	69336.105	1
Rollerblade X Fall	0.083	0.023	0.150	22918.337	1
Wheelchair X Fall	-0.083	-0.150	-0.023	22918.337	1
Rollerblade X Success	-0.056	-0.114	-0.006	37812.616	1
Wheelchair X Success	0.056	0.006	0.114	37812.616	1
SCI X Rollerblade X Safe Fail	-0.008	-0.102	0.021	13054.415	1

PHY X Rollerblade X Safe Fail	0.063	-0.009	0.131	6354.670	1
SKA X Rollerblade X Safe Fail	-0.005	-0.087	0.032	23452.806	1
SCI X Wheelchair X Safe Fail	0.008	-0.021	0.102	13054.415	1
PHY X Wheelchair X Safe Fail	-0.063	-0.131	0.009	6354.670	1
SKA X Wheelchair X Safe Fail	0.005	-0.032	0.087	23452.806	1
SCI X Rollerblade X Fall	0.004	-0.041	0.086	34057.818	1
PHY X Rollerblade X Fall	-0.002	-0.079	0.046	45198.751	1
SKA X Rollerblade X Fall	-0.001	-0.069	0.057	86213.572	1
SCI X Wheelchair X Fall	-0.004	-0.086	0.041	34057.818	1
PHY X Wheelchair X Fall	0.002	-0.046	0.079	45198.751	1
SKA X Wheelchair X Fall	0.001	-0.057	0.069	86213.572	1
SCI X Rollerblade X Success	0.003	-0.038	0.078	45425.738	1
PHY X Rollerblade X Success	-0.033	-0.113	0.015	9670.854	1
SKA X Rollerblade X Success	0.007	-0.026	0.094	16957.934	1
SCI X Wheelchair X Success	-0.003	-0.078	0.038	45425.738	1
PHY X Wheelchair X Success	0.033	-0.015	0.113	9670.854	1
SKA X Wheelchair X Success	-0.007	-0.094	0.026	16957.934	1

Table D.2 – Posterior distributions for the covariates for the Accuracies data for the 600-1800 ms Action Anticipation experiment

	Mode	HPDI		ESS	$\hat{R}$
Length	0.031	-0.089	0.158	123715.58	1
Video Ending: Safe Fail	-0.018	-0.152	0.100	122798.79	1
Video Ending: Fall	0.094	-0.028	0.226	105777.37	1
Video Ending: Success	-0.045	-0.168	0.084	122883.65	1
Video type: Rollerblade	0.062	-0.063	0.185	120107.65	1
Video type: Wheelchair	-0.024	-0.155	0.095	125128.02	1
Group: SCI	-0.021	-0.152	0.100	123100.23	1
Group: PHY	0.050	-0.080	0.171	117088.24	1
Group: SKA	0.009	-0.116	0.136	122706.58	1
Rollerblade X Safe Fail	0.036	-0.087	0.172	117735.72	1
Wheelchair X Safe Fail	-0.063	-0.197	0.063	100900.54	1
Rollerblade X Fall	0.016	-0.121	0.143	112661.49	1
Wheelchair X Fall	0.080	-0.046	0.222	83188.55	1
Rollerblade X Success	0.006	-0.120	0.137	122490.86	1
Wheelchair X Success	-0.044	-0.181	0.078	113861.64	1
SCI X Rollerblade	0.032	-0.099	0.161	121148.69	1

PHY X Rollerblade	0.043	-0.081	0.178	120301.74	1
SKA X Rollerblade	-0.015	-0.149	0.111	113980.79	1
SCI X Wheelchair	-0.049	-0.188	0.072	105681.46	1
PHY X Wheelchair	-0.006	-0.131	0.129	125000.00	1
SKA X Wheelchair	0.029	-0.097	0.164	113844.32	1
SCI X Safe Fail	0.008	-0.118	0.146	111078.65	1
PHY X Safe Fail	-0.027	-0.169	0.096	104796.25	1
SKA X Safe Fail	0.003	-0.133	0.129	123160.29	1
SCI X Fall	-0.050	-0.197	0.075	75734.66	1
PHY X Fall	0.089	-0.027	0.246	71592.86	1
SKA X Fall	0.050	-0.081	0.190	100364.77	1
SCI X Success	0.021	-0.108	0.155	110268.99	1
PHY X Success	-0.021	-0.156	0.107	117948.73	1
SKA X Success	-0.027	-0.174	0.090	114854.66	1
SCI X Rollerblade X Safe Fail	0.037	-0.096	0.180	107486.35	1
PHY X Rollerblade X Safe Fail	0.011	-0.132	0.144	123307.10	1
SKA X Rollerblade X Safe Fail	0.005	-0.142	0.133	122935.99	1
SCI X Wheelchair X Safe Fail	-0.030	-0.165	0.110	122814.95	1
PHY X Wheelchair X Safe Fail	-0.035	-0.178	0.098	117542.26	1
SKA X Wheelchair X Safe Fail	-0.001	-0.140	0.135	122990.56	1
SCI X Rollerblade X Fall	-0.037	-0.187	0.095	84541.84	1
PHY X Rollerblade X Fall	0.047	-0.083	0.199	96947.72	1
SKA X Rollerblade X Fall	0.003	-0.137	0.143	117304.51	1
SCI X Wheelchair X Fall	-0.008	-0.153	0.126	114041.74	1
PHY X Wheelchair X Fall	0.040	-0.086	0.194	108745.08	1
SKA X Wheelchair X Fall	0.047	-0.091	0.193	88920.82	1
SCI X Rollerblade X Success	0.032	-0.096	0.180	110411.32	1
PHY X Rollerblade X Success	-0.015	-0.151	0.125	116036.27	1
SKA X Rollerblade X Success	-0.009	-0.156	0.119	123620.14	1
SCI X Wheelchair X Success	-0.020	-0.157	0.118	122119.60	1
PHY X Wheelchair X Success	-0.011	-0.151	0.125	129594.28	1
SKA X Wheelchair X Success	-0.012	-0.156	0.119	122527.10	1

Table D.3 – Posterior distributions for the categorial factorsfor the Accuracies data for the 2400-3000 ms Action Anticipation experiment

	Mode	HPDI		ESS	$\hat{R}$
Intercept	3.161	2.885	3.447	20781.35	1
Group: SCI	-0.519	-0.878	-0.156	26123.32	1

Group: PHY	0.202	-0.142	0.559	50356.51	1
Group: SKA	0.310	-0.048	0.684	32853.46	1
Video type: Rollerblade	0.916	0.760	1.111	11081.20	1
Video type: Wheelchair	-0.916	-1.111	-0.760	11081.20	1
Video Ending: Safe Fail	-0.229	-0.453	0.003	15329.34	1
Video Ending: Fall	0.296	0.064	0.555	14625.69	1
Video Ending: Success	-0.082	-0.289	0.126	15607.00	1
SCI X Rollerblade	-0.264	-0.466	-0.079	13715.23	1
PHY X Rollerblade	0.122	-0.064	0.333	22066.75	1
SKA X Rollerblade	0.124	-0.075	0.355	19003.90	1
SCI X Wheelchair	0.264	0.079	0.466	13715.23	1
PHY X Wheelchair	-0.122	-0.333	0.064	22066.75	1
SKA X Wheelchair	-0.124	-0.355	0.075	19003.90	1
SCI X Safe Fail	-0.174	-0.426	0.051	19747.32	1
PHY X Safe Fail	0.136	-0.095	0.422	24563.21	1
SKA X Safe Fail	0.018	-0.240	0.289	29411.16	1
SCI X Fall	-0.091	-0.321	0.150	32715.13	1
PHY X Fall	0.118	-0.124	0.381	34580.56	1
SKA X Fall	-0.051	-0.318	0.227	30284.00	1
SCI X Success	0.262	0.038	0.505	19124.96	1
PHY X Success	-0.283	-0.535	-0.050	26142.26	1
SKA X Success	0.025	-0.232	0.281	29468.52	1
Rollerblade X Safe Fail	0.769	0.551	1.001	15585.11	1
Wheelchair X Safe Fail	-0.769	-1.001	-0.551	15585.11	1
Rollerblade X Fall	0.516	0.295	0.762	15525.82	1
Wheelchair X Fall	-0.516	-0.762	-0.295	15525.82	1
Rollerblade X Success	-1.306	-1.516	-1.100	14201.79	1
Wheelchair X Success	1.306	1.100	1.516	14201.79	1
SCI X Rollerblade X Safe Fail	-0.385	-0.654	-0.142	15377.89	1
PHY X Rollerblade X Safe Fail	0.227	-0.002	0.491	24456.30	1
SKA X Rollerblade X Safe Fail	0.133	-0.092	0.404	29107.83	1
SCI X Wheelchair X Safe Fail	0.385	0.142	0.654	15377.89	1
PHY X Wheelchair X Safe Fail	-0.227	-0.491	0.002	24456.30	1
SKA X Wheelchair X Safe Fail	-0.133	-0.404	0.092	29107.83	1
SCI X Rollerblade X Fall	0.050	-0.152	0.272	39306.53	1
PHY X Rollerblade X Fall	-0.164	-0.413	0.057	31176.63	1
SKA X Rollerblade X Fall	0.108	-0.123	0.373	30960.36	1
SCI X Wheelchair X Fall	-0.050	-0.272	0.152	39306.53	1
PHY X Wheelchair X Fall	0.164	-0.057	0.413	31176.63	1

SKA X Wheelchair X Fall	-0.108	-0.373	0.123	30960.36	1
SCI X Rollerblade X Success	0.328	0.102	0.583	14869.66	1
PHY X Rollerblade X Success	-0.054	-0.278	0.145	39403.76	1
SKA X Rollerblade X Success	-0.268	-0.522	-0.017	19334.40	1
SCI X Wheelchair X Success	-0.328	-0.583	-0.102	14869.66	1
PHY X Wheelchair X Success	0.054	-0.145	0.278	39403.76	1
SKA X Wheelchair X Success	0.268	0.017	0.522	19334.40	1

Table D.4 – Posterior distributions for the covariates for the Accuracies data for the 2400-3000 ms Action Anticipation experiment

	Mode	HPDI		ESS	$\hat{R}$
Length	0.460	0.042	0.887	238640.25	1
Video Ending: Safe Fail	0.148	-0.282	0.576	201923.55	1
Video Ending: Fall	0.269	-0.150	0.717	128925.21	1
Video Ending: Success	0.030	-0.394	0.460	236772.88	1
Video type: Rollerblade	0.171	-0.249	0.617	125548.60	1
Video type: Wheelchair	0.290	-0.149	0.699	241379.08	1
Group: SCI	0.042	-0.390	0.461	261195.23	1
Group: PHY	0.185	-0.239	0.619	212193.11	1
Group: SKA	0.236	-0.193	0.671	161237.07	1
Rollerblade X Safe Fail	-0.097	-0.557	0.352	98803.78	1
Wheelchair X Safe Fail	0.232	-0.185	0.683	207366.23	1
Rollerblade X Fall	0.121	-0.313	0.623	60987.51	1
Wheelchair X Fall	0.142	-0.306	0.572	161300.85	1
Rollerblade X Success	0.145	-0.310	0.572	153604.87	1
Wheelchair X Success	-0.093	-0.538	0.336	203074.29	1
SCI X Rollerblade	-0.163	-0.594	0.284	151045.36	1
PHY X Rollerblade	0.220	-0.234	0.667	113009.95	1
SKA X Rollerblade	0.111	-0.335	0.582	84309.95	1
SCI X Wheelchair	0.189	-0.230	0.633	253022.15	1
PHY X Wheelchair	-0.022	-0.461	0.409	218550.08	1
SKA X Wheelchair	0.115	-0.315	0.562	177347.46	1
SCI X Safe Fail	0.020	-0.438	0.433	240903.74	1
PHY X Safe Fail	0.126	-0.299	0.594	169284.45	1
SKA X Safe Fail	0.016	-0.454	0.443	158542.83	1
SCI X Fall	0.090	-0.330	0.555	212325.55	1
PHY X Fall	0.006	-0.413	0.485	166676.13	1
SKA X Fall	0.128	-0.306	0.604	129641.43	1

SCI X Success	-0.077	-0.508	0.365	249975.48	1
PHY X Success	0.025	-0.446	0.436	207031.42	1
SKA X Success	0.088	-0.355	0.546	152420.19	1
SCI X Rollerblade X Safe Fail	-0.070	-0.509	0.417	135623.82	1
PHY X Rollerblade X Safe Fail	-0.047	-0.548	0.438	89941.57	1
SKA X Rollerblade X Safe Fail	-0.041	-0.504	0.486	91692.05	1
SCI X Wheelchair X Safe Fail	0.047	-0.408	0.480	228718.58	1
PHY X Wheelchair X Safe Fail	0.178	-0.241	0.663	192451.41	1
SKA X Wheelchair X Safe Fail	-0.007	-0.449	0.457	191267.17	1
SCI X Rollerblade X Fall	0.085	-0.388	0.561	144903.06	1
PHY X Rollerblade X Fall	0.064	-0.388	0.593	97853.04	1
SKA X Rollerblade X Fall	-0.002	-0.522	0.477	84327.13	1
SCI X Wheelchair X Fall	0.016	-0.432	0.469	245941.12	1
PHY X Wheelchair X Fall	-0.058	-0.515	0.399	197828.87	1
SKA X Wheelchair X Fall	0.169	-0.287	0.635	166115.90	1
SCI X Rollerblade X Success	-0.177	-0.661	0.248	182062.22	1
PHY X Rollerblade X Success	0.182	-0.286	0.638	185796.36	1
SKA X Rollerblade X Success	0.163	-0.308	0.633	123858.99	1
SCI X Wheelchair X Success	0.120	-0.330	0.583	176142.98	1
PHY X Wheelchair X Success	-0.152	-0.634	0.278	220445.54	1
SKA X Wheelchair X Success	-0.051	-0.526	0.420	127418.01	1

Table D.5 – Posterior distributions for the Emotional Valence of the Stimuli data

	Mode	HPDI		ESS	$\hat{R}$
Intercept	19.952	14.563	25.599	125000.00	1
Emotion: Negative	-2.255	-2.902	-1.646	125000.00	1
Emotion: Neutral	2.255	1.646	2.902	125000.00	1
Video type: Rollerblades	-1.600	-2.214	-0.952	120086.82	1
Video type: Wheelchair	1.600	0.952	2.214	120086.82	1
Video Ending: Safe Fail	-0.560	-1.453	0.326	125000.00	1
Video Ending: Fall	6.323	5.385	7.176	122451.89	1
Video Ending: Success	-5.709	-6.642	-4.858	115598.50	1
Negative X Rollerblades	-0.536	-1.161	0.075	39802.35	1
Neutral X Rollerblades	0.536	-0.075	1.161	39802.35	1
Negative X Wheelchair	0.536	-0.075	1.161	39802.35	1
Neutral X Wheelchair	-0.536	-1.161	0.075	39802.35	1
Negative X Safe Fail	0.831	-0.004	1.733	118276.68	1
Neutral X Safe Fail	-0.831	-1.733	0.004	118276.68	1

Negative X Fall	1.785	0.877	2.641	87336.94	1
Neutral X Fall	-1.785	-2.641	-0.877	87336.94	1
Negative X Success	-2.624	-3.567	-1.761	62725.78	1
Neutral X Success	2.624	1.761	3.567	62725.78	1
Rollerblades X Safe Fail	0.617	-0.187	1.472	22233.50	1
Wheelchair X Safe Fail	-0.617	-1.472	0.187	22233.50	1
Rollerblades X Fall	-0.725	-1.652	0.077	16276.95	1
Wheelchair X Fall	0.725	-0.077	1.652	16276.95	1
Rollerblades X Success	0.049	-0.605	0.970	95962.82	1
Wheelchair X Success	-0.049	-0.970	0.605	95962.82	1
Negative X Rollerblades X Safe Fail	-0.003	-0.521	0.603	116871.90	1
Neutral X Rollerblades X Safe Fail	0.003	-0.603	0.521	116871.90	1
Negative X Wheelchair X Safe Fail	0.003	-0.603	0.521	116871.90	1
Neutral X Wheelchair X Safe Fail	-0.003	-0.521	0.603	116871.90	1
Negative X Rollerblades X Fall	-0.003	-0.650	0.491	77652.19	1
Neutral X Rollerblades X Fall	0.003	-0.491	0.650	77652.19	1
Negative X Wheelchair X Fall	0.003	-0.491	0.650	77652.19	1
Neutral X Wheelchair X Fall	-0.003	-0.650	0.491	77652.19	1
Negative X Rollerblades X Success	0.002	-0.502	0.630	107361.80	1
Neutral X Rollerblades X Success	-0.002	-0.630	0.502	107361.80	1
Negative X Wheelchair X Success	-0.002	-0.630	0.502	107361.80	1
Neutral X Wheelchair X Success	0.002	-0.502	0.630	107361.80	1

Table D.6 – Posterior distributions for the Kinematic data analyses

**Distance Covered**	Mode	HPDI		ESS	$\hat{R}$
Intercept	69.572	60.896	78.133	115103.25	1
Video type: Wheelchair	15.248	7.363	24.614	81404.18	1
Video type: Rollerblades	-15.248	-24.614	-7.363	81404.18	1
Video Ending: Safe Fail	-4.805	-16.037	6.855	99414.94	1
Video Ending: Fall	-13.000	-25.502	-1.745	58809.56	1
Video Ending: Success	18.244	5.564	30.308	44415.20	1
Wheelchair X Safe Fail	0.013	-10.220	8.277	104240.62	1
Rollerblades X Safe Fail	-0.013	-8.277	10.220	104240.62	1
Wheelchair X Fall	-0.251	-16.127	3.619	14372.63	1
Rollerblades X Fall	0.251	-3.619	16.127	14372.63	1
Wheelchair X Success	0.218	-2.879	17.426	11752.04	1
Rollerblades X Success	-0.218	-17.426	2.879	11752.04	1
**Distance Covered – covariation with Frame**					
Frame	0.066	0.001	0.125	122102.7	1
Video type: Wheelchair	0.034	-0.029	0.095	123312.9	1

Video type: Rollerblades	0.020	-0.039	0.085	121039.2	1
Video Ending: Safe Fail	0.007	-0.056	0.068	119383.7	1
Video Ending: Fall	0.026	-0.037	0.087	116399.6	1
Video Ending: Success	0.037	-0.027	0.097	118432.0	1
Wheelchair X Safe Fail	0.003	-0.060	0.065	120000.0	1
Rollerblades X Safe Fail	0.032	-0.030	0.094	118694.1	1
Wheelchair X Fall	0.003	-0.059	0.066	115967.3	1
Rollerblades X Fall	0.021	-0.042	0.083	116589.8	1
Wheelchair X Success	0.021	-0.042	0.084	114091.3	1
Rollerblades X Success	-0.013	-0.075	0.049	115114.8	1
**Right Elbow Angle**					
Intercept	1.719	1.648	1.796	120000.00	1
Video type: Wheelchair	-0.137	-0.214	-0.067	104118.81	1
Video type: Rollerblades	0.137	0.067	0.214	104118.81	1
Video Ending: Safe Fail	-0.202	-0.305	-0.096	78182.55	1
Video Ending: Fall	0.001	-0.102	0.101	120000.00	1
Video Ending: Success	0.203	0.096	0.306	77311.55	1
Wheelchair X Safe Fail	0.130	0.020	0.240	19496.42	1
Rollerblades X Safe Fail	-0.130	-0.240	-0.020	19496.42	1
Wheelchair X Fall	-0.017	-0.119	0.068	104581.18	1
Rollerblades X Fall	0.017	-0.068	0.119	104581.18	1
Wheelchair X Success	-0.100	-0.204	0.001	25499.04	1
Rollerblades X Success	0.100	-0.001	0.204	25499.04	1
**Right Elbow Angle – covariation with Frame**					
Frame	0.000	-0.014	0.014	120000.0	1
Video type: Wheelchair	0.000	-0.014	0.014	118581.9	1
Video type: Rollerblades	0.000	-0.014	0.014	120000.0	1
Video Ending: Safe Fail	0.001	-0.014	0.014	120000.0	1
Video Ending: Fall	0.000	-0.014	0.014	120000.0	1
Video Ending: Success	0.001	-0.014	0.014	118604.9	1
Wheelchair X Safe Fail	0.000	-0.014	0.014	120000.0	1
Rollerblades X Safe Fail	0.000	-0.014	0.014	120000.0	1
Wheelchair X Fall	0.001	-0.014	0.014	121288.4	1
Rollerblades X Fall	-0.001	-0.014	0.014	120000.0	1
Wheelchair X Success	-0.001	-0.014	0.014	120000.0	1
Rollerblades X Success	0.000	-0.014	0.014	120638.2	1
**Left Knee Angle**					
Intercept	0.233	0.193	0.267	126032.87	1
Video type: Wheelchair	-0.145	-0.181	-0.107	116248.11	1
Video type: Rollerblades	0.145	0.107	0.181	116248.11	1
Video Ending: Safe Fail	0.016	-0.026	0.067	76668.45	1
Video Ending: Fall	-0.008	-0.054	0.037	113172.79	1
Video Ending: Success	-0.007	-0.057	0.035	103192.81	1
Wheelchair X Safe Fail	0.003	-0.027	0.043	57465.41	1
Rollerblades X Safe Fail	-0.003	-0.043	0.027	57465.41	1
Wheelchair X Fall	-0.001	-0.038	0.030	94989.92	1
Rollerblades X Fall	0.001	-0.030	0.038	94989.92	1
Wheelchair X Success	-0.001	-0.038	0.031	97966.86	1

Rollerblades X Success	0.001	-0.031	0.038	97966.86	1
**Left Knee Angle – covariation with Frame**					
Frame	0.000	-0.014	0.014	122638.1	1
Video type: Wheelchair	0.000	-0.014	0.014	120000.0	1
Video type: Rollerblades	0.000	-0.014	0.015	120000.0	1
Video Ending: Safe Fail	0.000	-0.014	0.014	118651.4	1
Video Ending: Fall	0.000	-0.014	0.015	120000.0	1
Video Ending: Success	0.001	-0.014	0.014	120000.0	1
Wheelchair X Safe Fail	0.000	-0.014	0.015	120000.0	1
Rollerblades X Safe Fail	0.001	-0.014	0.014	120000.0	1
Wheelchair X Fall	0.000	-0.014	0.014	121615.8	1
Rollerblades X Fall	-0.001	-0.014	0.014	120000.0	1
Wheelchair X Success	0.001	-0.014	0.014	120000.0	1
Rollerblades X Success	0.000	-0.014	0.014	122227.8	1

References

Agresti, A. (2012). *Categorical Data Analysis* (Second). New Jersey: John Wiley & Sons, Inc. http://doi.org/10.1002/0471249688

Gelman, A., & Hill, J. (2006). *Data Analysis Using Regression and Multilevel/Hierarchical Models*.

Kruschke, J. K. (2011). *Doing Bayesian data analysis : a tutorial with R and BUGS* (Vol. 25). Burlington, MA: Academic Press.

Kruschke, J. K. (2014). *Doing Bayesian data analysis: A tutorial with R, JAGS, and Stan, second edition*. *Doing Bayesian Data Analysis: A Tutorial with R, JAGS, and Stan, Second Edition* (2nd ed.). Elsevier Inc. http://doi.org/10.1016/B978-0-12-405888-0.09999-2
